# Analysis of Genomic Regions Associated With Coronary Artery Disease Reveals Continent-Specific Single Nucleotide Polymorphisms in North African Populations

**DOI:** 10.2188/jea.JE20150034

**Published:** 2016-05-05

**Authors:** Daniela Zanetti, Marc Via, Robert Carreras-Torres, Esther Esteban, Hassen Chaabani, Fatima Anaibar, Nourdin Harich, Rachida Habbal, Noreddine Ghalim, Pedro Moral

**Affiliations:** 1 Department of Animal Biology-Anthropology, University of Barcelona, Barcelona, Spain; 2 Department of Psychiatry and Clinical Psychobiology and Institute for Brain, Cognition and Behavior (IR3C), Universitat de Barcelona, Barcelona, Spain; 3 Laboratory of Human Genetics and Anthropology, Faculty of Pharmacy, University of Monastir, 5000 Monastir, Tunisia; 4 Laboratoire des Sciences Anthropogénétiques, Faculté des Sciences, Université Chouaib Doukkali, El Jadida, Morocco; 5 Centre de Cardiologie, CHU IBN ROCHD, Casablanca, Morocco; 6 Laboratoire de Biochimie, Institut Pasteur du Maroc, Casablanca, Morocco

**Keywords:** CAD genetic risk, North Africa, SNPs, haplotype blocks, genetic association

## Abstract

**Background:**

In recent years, several genomic regions have been robustly associated with coronary artery disease (CAD) in different genome-wide association studies (GWASs) conducted mainly in people of European descent. These kinds of data are lacking in African populations, even though heart diseases are a major cause of premature death and disability.

**Methods:**

Here, 384 single nucleotide polymorphisms (SNPs) in the top four CAD risk regions (1p13, 1q41, 9p21, and 10q11) were genotyped in 274 case-control samples from Morocco and Tunisia, with the aim of analyzing for the first time if the associations found in European populations were transferable to North Africans.

**Results:**

The results indicate that, as in Europe, these four genetic regions are also important for CAD risk in North Africa. However, the individual SNPs associated with CAD in Africa are different from those identified in Europe in most cases (1p13, 1q41, and 9p21). Moreover, the seven risk variants identified in North Africans are efficient in discriminating between cases and controls in North African populations, but not in European populations.

**Conclusions:**

This study indicates a disparity in markers associated to CAD susceptibility between North Africans and Europeans that may be related to population differences in the chromosomal architecture of these risk regions.

## INTRODUCTION

Cardiovascular disease (CVD) continues to be the leading cause of mortality and morbidity in Western populations.^1^ According to World Health Organization data, 17.3 million people died from CVD in 2008, representing 30% of all global deaths (http://www.who.int/cardiovascular_diseases/about_cvd/en/). In North Africa and the Middle East, non-communicable diseases are an increasingly prevalent cause of premature death and disability (http://www.who.int/whr/2013/report/en/). For example, ischemic heart disease and stroke have increased by 44% and 35%, respectively, during the last 20 years.

The most common type of heart disease is coronary artery disease (CAD), which is a paradigm of complex disease, where environmental, lifestyle, and genetic factors interact to determine the clinical phenotype.^2^ In recent years, several genetic variants robustly associated with CAD have been detected, mainly in people of European descent (96%),^3^ through genome-wide association studies (GWASs). Previous surveys in non-European populations have suggested that markers associated in one population may not always easily translate to other populations. Associations found in Europeans must be investigated in other ethnic groups^3^ to assess the replicability of association signals or to detect new population-specific risk markers. For example, three variants in the major susceptibility gene (NOD2) for Crohn’s disease were associated with susceptibility in Europeans.^4^^,^^5^ However, the same variants did not show evidence of association in Morocco and Tunisia.^6^^,^^7^ Likewise, the genetic susceptibility alleles for glaucoma in European populations do not seem to play a substantial role in populations of African ancestry.^8^ On the contrary, a recent study affirmed that Europeans and North Africans share the same 13 risk markers for type 2 diabetes.^9^ For the most studied CAD risk regions, 9p21, the lack of replication of European association signals in African samples was previously reported for coronary artery calcification^10^ and ischemic stroke.^11^ These differences in association signals between different population groups raise intriguing questions, and an increase in the number of epidemiological studies in different populations is required not only to validate the known risk loci, but also to identify new population-specific susceptibility variants.

In this way, the present study deals with a fine analysis of the four most validated CAD risk regions, 1p13, 1q41, 9p21, and 10q11, which are included among the labeled “top 12 golden loci”,^2^ in a novel set of case-control samples from North Africa. The main goal is to check the level (genomic regions and/or individual sites) to which the CAD associations previously found in Europe may also be transferable to North Africa, specifically to people of Tunisian and Moroccan origin. To accomplish this goal, 384 single nucleotide polymorphism (SNPs) on those four genomic regions were genotyped in case-control samples from North Africa. The associations and trends detected in North African samples were compared with available data from southern Europe. Finally, the combined effects (risk score) of the associated markers found in North Africa, and their ability to discriminate between cases and controls, were assessed.

## MATERIALS AND METHODS

### Samples

A total of 142 cases and 132 controls from Morocco and Tunisia (52% males) were analyzed in the present study. The Moroccan samples consisted of 72 subjects from the area of Casablanca, along with 51 controls from the Doukkala-Abda region in west-central Morocco. The Tunisian case samples included 70 patients treated at the Department of Cardiology of the University Hospital Fattouma Bourguiba (Monastir, Tunisia). As a control group, 81 unrelated individuals, free from any CAD or related disorders, were randomly selected from the same large geographical area to which the patients belonged (the center of Tunisia). In both countries, CAD patients were diagnosed for ischemic heart disease complicated by myocardial infarction (MI), which was confirmed by electrocardiography and coronary angiography. All participants provided written informed consent, and the study was approved by the Ethics Committee of the University of Barcelona.

For comparative purposes, we used data from two southern European matched case-control samples (Milan, ATVB, in northern Italy, and Girona, Regicor, in north-eastern Spain) from the Myocardial Infarction Genetics (MIGen) Consortium,^12^ which was accessed through the database of Genotypes and Phenotypes (dbGAP; http://www.ncbi.nlm.nih.gov/gap). Baseline characteristics of the samples used in the study are shown in Table 1.

**Table 1.** Baseline characteristics of the samples

		Morocco	Tunisia	Milan	Girona
*N*	Cases	72	70	1693	312
	Controls	51	81	1668	317
Mean Age (SD)	Cases	56.5 (10.8)	40.3 (10.1)	39.4 (4.9)	45.9 (5.9)
	Controls	23.0 (1.5)	23.8 (3.4)	39.3 (5.0)	46.0 (5.6)
% Males	Cases	68.0	55.7	88.3	79.5
	Controls	54.9	29.6	88.3	78.5

SD, standard deviation.

### Polymorphisms and genotyping

Genomic DNA was extracted from blood cells using a Blood Midi kit (Omega Biotek, Norcross, GA, USA) in accordance with the manufacturer’s procedures. DNA samples were genotyped for a set of 384 SNPs using a Custom GoldenGate Panel (Illumina Inc., San Diego, CA, USA). Out of the 384 SNPs, 61 are located in the 1p13.3 chromosomal region, which spans 150 kb and includes the *CELSR2*, *PSRC1*, *MYBPHL*, and *SORT1* genes; 38 SNPs are in 1q41, which spans 100 kb and includes the *TAF1A*, *MIA3*, and *AIDA* genes; 159 SNPs are in 9p21, which spans 300 kb and comprises the *CDKN2A* and *CDKN2B* genes; and finally, 126 SNPs are in the 10q11 chromosomal region, which spans 200 kb and includes the *CXCL12* gene.

SNPs were selected as a representative set of the common variation in the four genomic regions according to the following criteria: i) average coverage of 1 SNP every 1.5 kb; ii) minor allele frequency (MAF) higher than 0.05 in European (CEU) HapMap populations; iii) avoiding markers in tight linkage disequilibrium (LD) (*r*^2^ > 0.8); and iv) giving priority to markers previously reported as associated with CAD.^13^ These criteria were applied to give preference to tag SNPs.

Genotype data coming from the MIGen samples were generated in the corresponding original projects using the Affymetrix 6.0 GeneChip (Affymetrix, Inc., Santa Clara, CA, USA).^12^

### Statistical analyses

Genotyping rate per SNP and individual, Hardy-Weinberg equilibrium (HWE), cryptic relatedness, MAF, and LD were checked using PLINK version 1.07 (Center for Human Genetic Research, Massachusetts General Hospital, and the Broad Institute of Harvard & Massachusetts Institute of Technology, Cambridge, MA, USA).^14^ Individuals with more than 5% of missing genotypes were excluded. SNPs with genotyping rate lower than 0.95 or with a MAF lower than 5% were also removed from the analyses.

In order to have the same genetic information, several SNPs not originally genotyped in MIGen samples were imputed simultaneously for cases and controls using MACH 1.0 software (Department of Biostatistics and Center for Statistical Genetics, University of Michigan, Ann Arbor, MI, USA).^15^ Phased chromosomes from the Tuscan (TSI) samples from the 1000 Genomes Project^16^ were used as a reference panel. A standard single-step imputation approach, with 200 rounds of Markov chain iterations, was used to estimate the crossover maps, error rate maps, and all missing genotypes.

Associations were tested by logistic-regression models adjusted for gender. Permutation tests (1000 permutations) were applied to assess the statistical significance (*P* ≤ 0.05) of the regression models. Permutation procedures provide a computationally intensive approach to empirically generating significance levels. Associations and permutations were calculated using PLINK.^14^ In the case of the European case-control samples, logistic-regression models adjusted for gender were calculated using both the allelic dosage approach, which accounts for imputation uncertainty, and the most likely genotype approach. The mach2dat software version 119 (Department of Biostatistics and Center for Statistical Genetics, University of Michigan)^17^ was used for the dosage approach. Statistical power of the North African samples was checked by the QUANTO software version 1.2 (University of Southern California, Los Angeles, CA, USA).^18^

Haplotype structure and LD pattern analyses were performed by HaploView version 4.2 (Broad Institute)^19^ using the algorithm proposed by Gabriel et al.^20^ Monte Carlo statistical significance was assessed for each chromosomal region in order to evaluate differences between populations in regional LD structure based on the *r*^2^. This analysis was carried out using 100 random individuals for each different population group (North Africa and southern Europe) using VarLD software version 1.0 (National University of Singapore, Singapore).^21^

Genetic differentiation between Moroccans and Tunisians was calculated by means of a Fisher’s exact probability test through Genepop version 4.2 (Laboratiore de Genetique et Environment, Montpellier, France).^22^

Considering that sample sizes of cases and controls from Morocco and Tunisia were not very large, three random effect meta-analyses were performed to provide pooled allelic odd ratios (ORs) with: i) samples from North Africa, ii) samples from southern Europe, and iii) both populations together, paying special attention to markers found as significantly associated in North Africa. Besides the statistical significance, an association was considered positive if the distribution pattern of the risk allele frequency was similar in the different case-control groups. The meta-analyses and the degree of heterogeneity (I^2^) in the different groups analyzed were calculated by means of the *Metafor* R package (R Foundation for Statistical Computing, Vienna, Austria).^23^ The I^2^ index describes the percentage of total variation across studies due to true heterogeneity rather than chance. A value of 0% indicates no observed heterogeneity, and larger values show increasing heterogeneity.

The joint effect of multiple risk SNPs (risk score) was explored by a logistic-regression model that included the number of risk alleles as an independent variable. The increase in risk was quantified by adding supplementary risk alleles according to results of the North African meta-analysis. In this case, homozygotes for the protective allele were coded as 0, heterozygotes as 1, and homozygotes for the risk allele were coded as 2. Additionally, the overall score (ie, the number of risk alleles) was calculated for each individual and compared between cases and controls. The ability of this model to discriminate between patients and healthy controls was evaluated through receiver operating characteristic (ROC) curves using logistic-regression models. The area under the ROC curve (AUC) was calculated as a measure of discriminative accuracy. The risk score and the ROC curve were calculated using the *PredictABEL* R package^24^ (R Foundation for Statistical Computing).

A flowchart of all the analyses performed is included as eFigure 1.

## RESULTS

### Genotyping, quality control, and imputation

According to the above criteria, the initial design included 1 SNP every 1.8 Kb, and the genotyping rate for the 384 SNPs initially tested was 95.7%. However, 15 SNPs were not successfully genotyped, 1 SNP showed a significant departure from Hardy-Weinberg equilibrium after Bonferroni correction, and 19 SNPs failed the frequency test (MAF <0.05); these were excluded from the study. Thus, a total of 349 markers were included in the analyses after quality control: 57 SNPs in 1p13, 36 in 1q41, 148 in 9p21, and 108 in 10q11 (a detailed list can be found in eTable 1). Moreover, one individual was removed for low genotyping rate (>5% of missing genotypes). The statistical power of the Moroccan and Tunisian samples was low when the two samples were analyzed separately. For example, a SNP with a MAF of 0.25 had a statistical power of 37.7% and 45.8% for Moroccans and Tunisians, respectively, to detect an OR of 1.6 with a type I error rate of 0.05. However, when we considered both samples together, the statistical power substantially increased to 70.1% under the same assumptions as above.

Concerning comparison data from MIGen project, genotyping status and imputation quality index (*r*^2^) are presented in eTable 1. One hundred and seventy-four out of the 349 SNPs were imputed, and only 14 of them showed an imputation quality lower than 0.3 in at least one case-control sample. Therefore, 335 SNPs were considered consistent for this analytical epidemiologic analysis. Most of the imputed SNPs (79%) showed high accuracy (*r*^2^ > 0.75), indicating good quality of the imputation performed. After quality control per individual, a total of 4979 case-control samples from Europe were used for comparisons, with 3352 individuals from the ATVB (Italy) and 627 from the Regicor (Spain) studies.

### Marker association analyses

A total of 5 SNPs were associated with CAD in Morocco (2 in the 1p13 chromosomal region and 1 in each one of the other three regions), whereas 26 were associated with CAD in Tunisia (5 in the 1p13 region, 7 in the 9p21, and 14 in the 10q11 region). Logistic regression results and genetic effects of these SNPs are shown in eTable 2. None of these SNPs was associated independently in Morocco and Tunisia, but most of them (24 out of 31) showed the same trends in the two African populations (ie, OR values higher or lower than 1).

Preliminary comparisons of the results in North Africa with the two European samples from the MIGen Consortium pointed out a low replicability of association signals between both groups of populations (eTable 2). For example, 12 out of 26 of the SNPs associated in the Tunisian sample were also associated in the ATVB study, but 4 of them showed an opposite effect.

An exact test indicated consistent genetic similarity between the two North African countries for both cases (*P* = 0.248) and controls (*P* = 0.927). In the meta-analysis of North African samples, seven SNPs showed significant associations with a remarkably low heterogeneity (I^2^ = 0%) (Table 2), with two corresponding to the 9p21 region and five corresponding to the 10q11 region. All of these SNPs were located in intergenic regions. In the 1p13 and 1q41 regions, no association was detected.

**Table 2.** Results of the three meta-analyses performed in North Africans, South Europeans, and merging both continental groups

CHR	BP	SNP	North Africa				South Europe				All groups			
		
			*P*	OR	CI	I^2^	*P*	OR	CI	I^2^	*P*	OR	CI	I^2^
**9**	22136489	rs1333051	0.016	0.470	(0.250–0.870)	0	0.726	1.020	(0.900–1.160)	0	0.410	0.840	(0.560–1.260)	74
**9**	22191189	rs828576	0.038	1.530	(1.020–2.290)	0	0.185	0.940	(0.860–1.030)	0	0.784	1.030	(0.850–1.230)	39
**10**	44730995	rs7907961	0.019	1.870	(1.110–3.140)	0	0.851	1.010	(0.910–1.120)	0	0.376	1.070	(0.920–1.260)	21
**10**	44786364	rs800314	0.013	0.380	(0.170–0.810)	0	0.410	0.870	(0.630–1.210)	51	0.152	0.740	(0.490–1.120)	56
**10**	44855663	rs266105	0.007	2.180	(1.240–3.850)	0	0.729	0.980	(0.860–1.110)	0	0.245	1.260	(0.850–1.860)	70
**10**	44856370	rs266103	0.019	1.890	(1.110–3.220)	0	0.601	0.970	(0.850–1.100)	0	0.369	1.150	(0.850–1.54)	53
**10**	44861220	rs7918568	0.047	1.600	(1.010–2.530)	0	0.300	1.060	(0.950–1.180)	0	0.144	1.080	(0.970–1.200)	0

CI, confidence interval; I^2^, index to quantify the % of true heterogeneity (total heterogeneity/total variability); OR, odds ratio.

The potential population specificity of the seven risk variants detected in North Africa was assessed by comparison with the results of the meta-analyses carried out in the European populations. None of these seven SNPs showed significant associations in southern European populations (Table 2). The heterogeneity among the southern European samples was remarkably low, except for the rs800314 marker (I^2^ = 51%).

The third meta-analysis performed using the North Africans and the southern Europeans case-control samples did not show significant associations for the seven SNPs previously identified in the African meta-analyses. The heterogeneity among African and European samples was medium to high (I^2^ from 21% to 74%), except for the rs7918568 marker (I^2^ = 0%) (Table 2).

### Haplotype analyses

Haplotype structure was analyzed to better understand the patterns of associations observed in these two different populations groups. As expected, different patterns of haplotype blocks were identified in North Africa and southern Europe (LD patterns and haplotype block structure are plotted in eFigure 2A, eFigure 2B, eFigure 2C, and eFigure 2D). In general, a higher number of haplotype blocks in North Africans compared to southern Europeans were observed in all four chromosomal regions: 6 versus 2 blocks in 1p13; 4 versus 2 in 1q41; 23 versus 21 in 9p21; and 12 versus 10 in 10q11. The two population groups showed statistically significant differences in LD patterns for all four chromosomal regions (*P* < 0.001 for the 1p13 and 9p21 regions, and *P* < 0.05 for the 1q41 and 10q11 genomic regions). The comparison of the haplotype blocks that included the seven African risk SNPs indicated that these risk variants are not located in the same haplotype blocks in African and European individuals (eFigure 2C and eFigure 2D).

### Risk scores

**Figure 1.**
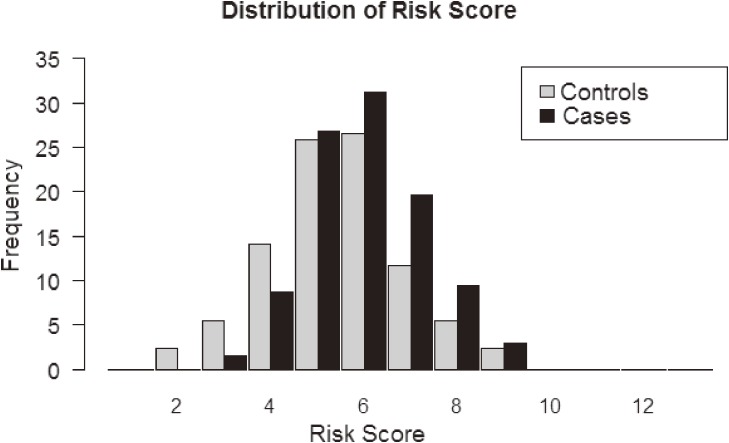
Distribution of North African individuals according to the number of risk alleles in the sample of cases and controls analyzed.

**Figure 2.**
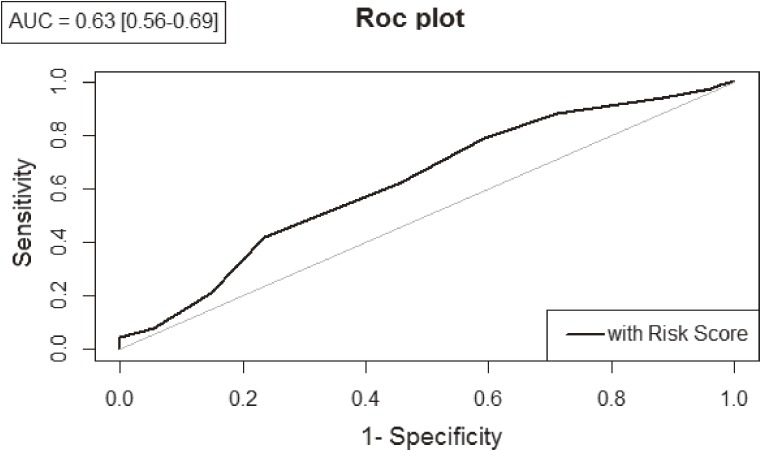
Receiver operating characteristic (ROC) curves in North Africa. AUC, Area Under the ROC Curve with 95% confidence interval.

**Figure 3.**
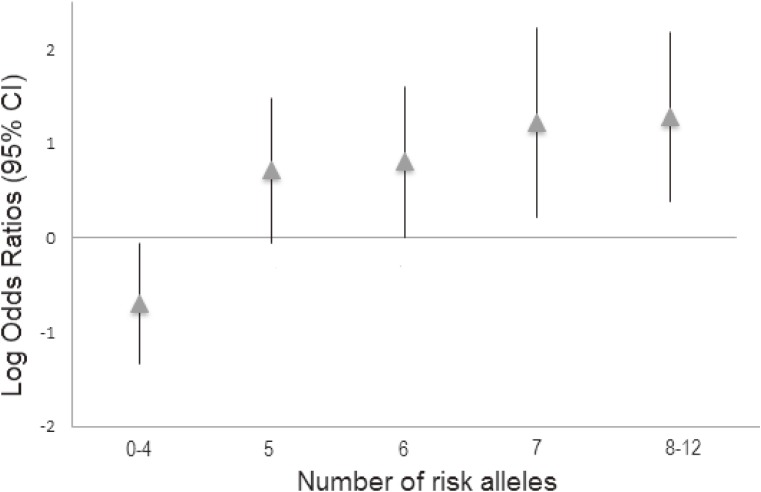
Logarithm of the odds ratios with 95% confidence interval for CAD (adjusted by gender) in North African individuals carrying increasing numbers of risk alleles.

The genomic risk-score was calculated using the information of SNPs associated in the North African meta-analysis. After applying LD pruning criteria in both European and North African samples (*r*^2^ < 0.4), six SNPs (making possible risk scores from 0 to 12 risk alleles) were considered in the analysis (eTable 3). In North Africa, the distribution of the risk score between patients and healthy individuals showed significant differences (Figure 1). A total of 32% of cases carried 6 risk alleles, whereas only 26% of controls showed the same amount of risk alleles. The risk score applied in North Africa showed a remarkable ability to discriminate between individuals with and without the disease (AUC = 0.63; 95% CI, 0.56–0.69) (Figure 2). In addition, the risk for CAD in North Africa increases with the number of risk alleles included in the risk score. The susceptibility to develop CAD in North Africans shows a significant value from the group that had 6 risk alleles (*P* = 0.04) and, as the number of risk alleles increases, the OR value also increases, reaching a maximum for the individuals with 8–12 risk alleles (Figure 3). Comparatively, this risk score had remarkably lower ability to predict CAD risk in southern Europeans than in North Africans (AUC = 0.52; 95% CI, 0.50–0.54) (eFigure 3).

## DISCUSSION

This study provides novel genetic data of epidemiological interest in North Africa. The genomic variation of four CAD risk regions (1p13, 1q41, 9p21, and 10q11) was analyzed through the genotyping of 384 SNPs in two North African case-control samples, specifically from Morocco and Tunisia. Previous studies of these genomic regions were based on populations of European descent. To the best of our knowledge, these are the first results in populations from Africa. Consequently, this study aimed to assess whether findings about CAD risk variants in European populations can also be extended to North Africans.

We found significant association signals in the four genomic regions analyzed also in Moroccan and Tunisian samples. However, in the meta-analyses performed with the African case-control samples, only the 9p21 and 10q11 regions showed association signals. None of the North African risk SNPs showed association in European samples. In addition, the calculated risk model had a remarkable ability to discriminate cases and controls in Africa (AUC = 0.63) but not in Europe (AUC = 0.52). These arguments suggest the existence of continental-specific CAD risk variants in these chromosomal regions.

### Continent-specific risk markers within global risk regions

Among the several CAD risk loci identified so far, the 9p21 locus has a prominent position because of the impressive robustness of the association results.^25^^–^^27^ In the North African meta-analysis, two SNPs in this genomic region showed association with CAD. A detailed analysis of the literature revealed that the rs1333051 marker was previously associated with type 2 diabetes in Mexican Americans.^28^ The 10q11 genomic region shows the highest number of associated SNPs in North Africa (5 out of 7 of the associated SNPs). None of these five SNPs was found to be associated with CAD in previous studies.

Regarding the logistic association performed in the four populations analyzed (Morocco, Tunisia, Italy, and Spain), the associated SNPs were not the same across populations, even though association signals were present in all of the regions studied. Of note, some associated SNPs are close to SNPs associated in other populations, while other SNPs are population-specific (ie, physically distant or not in LD with SNPs associated in other populations).

The number of SNPs associated with risk of CAD in southern Europe is considerably larger than in North Africa, as expected from the much larger sample size (and statistical power) of the European dataset. The ability of the limited sample size to reach the significance threshold of the North African samples may be the cause of the low number of associations found in Africa and the lack of replication in African samples of most associations found in Europe. Still, the risk SNPs found in North Africa but not replicated in Europe underline the possible role of the genomic architecture in these results. Statistical power calculations indicate reduced power of the North African case-control samples to detect small genetic effects, but adequate power to detect a risk with an OR ≥1.6. Although sample size biases cannot be fully discounted, the SNPs associated in Africa but not in Europe show an adequate statistical consistency according to the permutation tests performed.

The significant differences (*P* < 0.05) in the LD and the haplotype patterns in the two population groups (eFigure 2A, eFigure 2B, eFigure 2C, and eFigure 2D) confirm the heterogeneity in the genomic structure between North Africans and Europeans. This general trend of lower haplotype diversities and higher LD values when the geographic distance from Africa increases has been previously observed.^29^^,^^30^ Further, the haplotype blocks containing risk SNPs differ between North Africa and southern Europe. This situation is especially evident in the 10q11 region, where three out of the five associated SNPs are not located in any block in North Africa, while in Europe only one of these SNPs is not located in a block (eFigure 2D). Differences in allele frequency and LD patterns may be related to the observed differences in the association tests. Higher LD (illustrated by the different number of haplotype blocks) in populations of European origin compared to Africans reduces the likelihood of finding a substantial number of associated SNPs in North Africa.^31^ African populations are genetically more diverse than European and Asian populations, a finding that is in agreement with the Recent-African-Origin hypothesis.^32^ Consequently, replication studies across European populations have been largely successful because of the general genetic similarity across European populations. A failure to replicate an association signal in Africa may be influenced by variations in LD patterns between European and African populations.^31^ Low levels of LD reduce the correlation among casual variants and nearby SNPs. As a consequence, the probability of detecting association signals is lower among Africans than in Europeans if the causal variants are not assayed directly.

The potential existence of ethnicity-specific CAD risk markers is not an isolated case; rather, such risk markers are common in complex diseases. A recent study detected significant differences in association signals among African and European populations screening the catalog of all published GWAS curated at the National Human Genome Research Institute website.^33^ This work stressed a modest correlation in the risk allele frequencies between Europeans and Africans. The point estimates of risk were opposite in direction or differed more than 2-fold in 79% of the comparisons between European and African groups. Along with this fact, the under-representation of non-European population in GWAS suggests the need to extend epidemiological studies to a much broader ensemble of populations, including ethnic minorities.^3^

### Conclusion

This study shows that in North African populations, as in European populations, the regions 1p13, 1q41, 9p21, and 10q11 contain genetic risk variants associated with CAD. However, Europeans and North Africans do not always share the same risk variants. The differences obtained in CAD risk variants between Europeans and North Africans suggest that findings from one population cannot be directly applied to populations from other continents. That is, differences in the genomic architecture due to different demographic histories should be taken into account in epidemiological studies of complex disorders. This preliminary evidence from four CAD risk genomic regions in North African populations needs to be extended in additional larger analyses.

## ONLINE ONLY MATERIALS

eTable 1. Genomic location of the genetic variants, genotyping, and imputation details.

eTable 2. Case-control allelic frequencies and association parameters adjusted for gender in the genotype and imputed case-control samples.

eTable 3. Genomic location of the 6 North African SNPs used to calculate the risk score in the case-control samples.

eFigure 1. Flowchart of all the analyses performed.

eFigure 2A. Linkage disequilibrium patterns and haplotype block structure observed in North Africa and in southern Europe for the region 1p13.

eFigure 2B. Linkage disequilibrium patterns and haplotype block structure observed in North Africa and in southern Europe for the region 1q41.

eFigure 2C. Linkage disequilibrium patterns and haplotype block structure observed in North Africa and in southern Europe for the region 9p21.

eFigure 2D. Linkage disequilibrium patterns and haplotype block structure observed in North Africa and in southern Europe for the region 10q11.

eFigure 3. Receiver operating characteristic (ROC) curves in southern Europe.
