# Supplementary material for: Analysis of Genomic Regions Associated With Coronary Artery Disease Reveals Continent-Specific Single Nucleotide Polymorphisms in North African Populations
Source: J Epidemiol. 2016 May 5;26(5):264–71. doi: 10.2188/jea.JE20150034 (PMC4848325; doi:10.2188/jea.JE20150034)
Supplement: eTable 1. [file je-26-264-s001.pdf]

eTable 1. Genomic location of the genetic variants, genotyping and imputation details

							ATVB				Regicor			
CHR	BP	SNP	Region	Status	Al1	Al2	FreqAl1	MAF	Quality	Rsq	FreqAl1	MAF	Quality	Rsq
1	109804646	rs4970833	1p13	IMPUT	A	G	0.529	0.471	0.565	0.244	0.517	0.483	0.569	0.251
1	109806313	rs653635	1p13	IMPUT	T	C	0.919	0.082	0.858	0.265	0.909	0.091	0.850	0.308
1	109807283	rs6657811	1p13	IMPUT	A	T	0.907	0.093	0.924	0.646	0.901	0.099	0.914	0.616
1	109808117	rs608196	1p13	IMPUT	C	T	0.918	0.082	0.859	0.268	0.909	0.091	0.850	0.311
1	109810981	rs17035630	1p13	IMPUT	G	A	0.914	0.086	0.835	0.057	0.919	0.081	0.844	0.049
1	109813719	rs17035665	1p13	IMPUT	C	T	0.802	0.198	0.736	0.277	0.796	0.204	0.724	0.263
1	109814880	rs4970834	1p13	IMPUT	C	T	0.879	0.121	0.960	0.848	0.865	0.135	0.960	0.866
1	109815252	rs611917	1p13	IMPUT	A	G	0.755	0.245	0.837	0.649	0.734	0.266	0.834	0.669
1	109817590	rs12740374	1p13	IMPUT	G	T	0.819	0.181	0.997	0.990	0.806	0.194	0.998	0.993
1	109818306	rs629301	1p13	GENOT	T	G	0.820	0.181	1	1	0.806	0.194	1	1
1	109818530	rs646776	1p13	IMPUT	T	C	0.819	0.181	0.999	0.996	0.806	0.194	0.999	0.998
1	109820919	rs17035949	1p13	GENOT	T	G	0.948	0.052	1	1	0.946	0.054	1	1
1	109821307	rs583104	1p13	GENOT	T	G	0.806	0.194	1	1	0.790	0.210	1	1
1	109821511	rs602633	1p13	IMPUT	G	T	0.823	0.177	0.970	0.919	0.809	0.191	0.966	0.915
1	109822166	rs599839	1p13	GENOT	A	G	0.807	0.193	1	1	0.790	0.210	1	1
1	109822509	rs14000	1p13	GENOT	T	C	0.889	0.111	1	1	0.881	0.119	1	1
1	109827253	rs672569	1p13	GENOT	G	A	0.892	0.108	1	1	0.882	0.118	1	1
1	109832283	rs655246	1p13	IMPUT	G	A	0.568	0.432	0.656	0.394	0.555	0.445	0.649	0.391
1	109833187	rs17584208	1p13	GENOT	G	A	0.956	0.044	1	1	0.948	0.053	1	1
1	109834938	rs17645031	1p13	IMPUT	C	T	0.956	0.044	0.998	0.980	0.948	0.052	0.998	0.977
1	109835757	rs17645143	1p13	IMPUT	T	C	0.680	0.320	0.665	0.338	0.684	0.316	0.661	0.332
1	109838918	rs629001	1p13	IMPUT	T	C	0.960	0.040	0.978	0.789	0.950	0.051	0.971	0.800
1	109839738	rs3850615	1p13	IMPUT	C	A	0.921	0.079	0.905	0.396	0.913	0.087	0.901	0.428
1	109840629	rs600806	1p13	GENOT	G	A	0.219	0.219	1	1	0.239	0.239	1	1
1	109842271	rs680434	1p13	IMPUT	C	T	0.263	0.263	0.939	0.851	0.278	0.278	0.941	0.857
1	109843775	rs11583969	1p13	GENOT	T	C	0.049	0.049	1	1	0.064	0.064	1	1
1	109844532	rs652651	1p13	IMPUT	A	G	0.242	0.242	0.963	0.921	0.261	0.261	0.961	0.919
1	109846278	rs407102	1p13	IMPUT	C	T	0.253	0.253	0.948	0.872	0.268	0.268	0.949	0.874
1	109848726	rs592107	1p13	IMPUT	A	T	0.253	0.253	0.948	0.870	0.268	0.268	0.949	0.873
1	109851127	rs444387	1p13	IMPUT	A	G	0.252	0.252	0.922	0.813	0.269	0.269	0.924	0.821
1	109856306	rs464218	1p13	IMPUT	G	A	0.421	0.421	0.968	0.931	0.453	0.453	0.968	0.929
1	109857815	rs17585355	1p13	GENOT	A	C	0.970	0.030	1	1	0.965	0.035	1	1
1	109858119	rs370088	1p13	IMPUT	T	C	0.247	0.247	0.932	0.834	0.263	0.263	0.934	0.843
1	109864269	rs3853500	1p13	IMPUT	T	C	0.245	0.245	0.933	0.837	0.261	0.261	0.937	0.847
1	109866569	rs3768496	1p13	IMPUT	T	A	0.249	0.249	0.981	0.948	0.267	0.267	0.983	0.954
1	109871787	rs10745352	1p13	IMPUT	T	C	0.249	0.249	0.982	0.951	0.267	0.267	0.984	0.956
1	109877506	rs10745353	1p13	IMPUT	A	G	0.248	0.248	0.985	0.961	0.267	0.267	0.986	0.963
1	109879549	rs4603158	1p13	IMPUT	C	T	0.248	0.248	0.987	0.964	0.266	0.266	0.988	0.966
1	109880721	rs11102972	1p13	IMPUT	C	T	0.209	0.209	0.939	0.829	0.222	0.222	0.942	0.838
1	109882250	rs10858084	1p13	IMPUT	T	A	0.248	0.248	0.992	0.978	0.266	0.266	0.990	0.973
1	109884775	rs2228604	1p13	IMPUT	T	G	0.247	0.247	0.995	0.986	0.266	0.266	0.996	0.990
1	109887191	rs4970843	1p13	GENOT	T	C	0.455	0.455	1	1	0.502	0.498	1	1
1	109891423	rs3879448	1p13	GENOT	G	C	0.255	0.255	1	1	0.279	0.279	1	1
1	109894693	rs7536292	1p13	GENOT	T	C	0.826	0.174	1	1	0.810	0.190	1	1
1	109900707	rs1030522	1p13	IMPUT	C	G	0.246	0.246	0.994	0.983	0.266	0.266	0.995	0.985
1	109904971	rs3879449	1p13	IMPUT	A	T	0.246	0.246	0.992	0.978	0.266	0.266	0.993	0.981
1	109910013	rs11581665	1p13	IMPUT	T	C	0.135	0.135	0.994	0.973	0.145	0.145	0.995	0.978
1	109915075	rs10858086	1p13	IMPUT	C	A	0.246	0.246	0.991	0.975	0.266	0.266	0.992	0.978
1	109922377	rs1149175	1p13	GENOT	A	G	0.134	0.134	1	1	0.144	0.144	1	1
1	109923677	rs12037569	1p13	IMPUT	G	T	0.863	0.137	0.907	0.661	0.851	0.149	0.893	0.651
1	109926828	rs10858089	1p13	IMPUT	T	C	0.246	0.246	0.993	0.980	0.265	0.265	0.993	0.981
1	109930852	rs4970752	1p13	IMPUT	C	T	0.246	0.246	0.993	0.982	0.266	0.266	0.994	0.983
1	109931908	rs10745354	1p13	IMPUT	C	T	0.246	0.246	0.994	0.982	0.266	0.266	0.994	0.983
1	109935427	rs3768494	1p13	IMPUT	A	G	0.246	0.246	0.995	0.985	0.266	0.266	0.995	0.985
1	109941133	rs1880670	1p13	IMPUT	C	T	0.246	0.246	0.998	0.994	0.266	0.266	0.998	0.994
1	109943893	rs10858092	1p13	GENOT	C	T	0.246	0.246	1	1	0.266	0.266	1	1
1	109949008	rs4120621	1p13	IMPUT	A	G	0.249	0.249	0.958	0.889	0.267	0.267	0.981	0.947
1	222751034	rs2936033	1q41	IMPUT	C	T	0.721	0.279	0.772	0.494	0.725	0.276	0.771	0.491
1	222759007	rs3008647	1q41	IMPUT	C	T	0.841	0.159	0.973	0.902	0.841	0.159	0.976	0.913
1	222761771	rs2270705	1q41	IMPUT	A	G	0.876	0.124	0.767	0.062	0.878	0.123	0.770	0.053
1	222762316	rs2936027	1q41	IMPUT	C	T	0.886	0.115	0.998	0.991	0.866	0.134	0.999	0.995
1	222762709	rs17464857	1q41	GENOT	T	G	0.839	0.161	1	1	0.839	0.161	1	1
1	222762773	rs2936041	1q41	GENOT	A	T	0.841	0.159	1	1	0.839	0.161	1	1
1	222763026	rs4846767	1q41	IMPUT	T	C	0.771	0.229	0.856	0.661	0.750	0.251	0.858	0.675
1	222763215	rs3008650	1q41	GENOT	C	G	0.882	0.118	1	1	0.858	0.142	1	1
1	222763661	rs2936040	1q41	GENOT	T	A	0.886	0.115	1	1	0.866	0.134	1	1

1	222764791	rs3008653	1q41	GENOT	C	G	0.839	0.161	1	1	0.839	0.161	1	1
1	222766609	rs3008654	1q41	IMPUT	G	A	0.869	0.131	0.909	0.702	0.869	0.131	0.912	0.708
1	222769593	rs1995152	1q41	IMPUT	A	C	0.886	0.115	0.998	0.988	0.866	0.134	0.998	0.991
1	222770703	rs1909194	1q41	GENOT	A	G	0.839	0.161	1	1	0.839	0.161	1	1
1	222772139	rs3002130	1q41	GENOT	T	C	0.839	0.162	1	1	0.839	0.161	1	1
1	222788062	rs3002142	1q41	GENOT	T	C	0.876	0.124	1	1	0.880	0.120	1	1
1	222790366	rs904323	1q41	IMPUT	G	A	0.834	0.166	0.865	0.599	0.823	0.177	0.852	0.579
1	222795118	rs17163303	1q41	GENOT	G	T	0.932	0.068	1	1	0.926	0.074	1	1
1	222798965	rs17011666	1q41	IMPUT	A	G	0.795	0.205	0.959	0.883	0.788	0.212	0.955	0.880
1	222802376	rs2936052	1q41	IMPUT	A	G	0.822	0.179	0.963	0.886	0.817	0.183	0.959	0.878
1	222804046	rs3008621	1q41	IMPUT	G	A	0.856	0.144	0.996	0.984	0.846	0.155	0.997	0.989
1	222807013	rs3002145	1q41	GENOT	C	T	0.855	0.145	1	1	0.845	0.155	1	1
1	222809616	rs1391557	1q41	IMPUT	C	T	0.824	0.176	0.998	0.993	0.819	0.181	0.997	0.992
1	222813753	rs2133188	1q41	GENOT	G	T	0.824	0.176	1	1	0.818	0.182	1	1
1	222814442	rs2133189	1q41	GENOT	T	C	0.729	0.271	1	1	0.716	0.284	1	1
1	222820639	rs17163358	1q41	GENOT	A	G	0.729	0.271	1	1	0.717	0.283	1	1
1	222821709	rs17531063	1q41	IMPUT	T	G	0.933	0.068	1.000	0.997	0.927	0.073	1.000	0.997
1	222823529	rs17465637	1q41	GENOT	C	A	0.731	0.269	1	1	0.717	0.283	1	1
1	222825088	rs17011681	1q41	IMPUT	G	C	0.730	0.270	1.000	0.999	0.716	0.284	0.999	0.998
1	222826481	rs2291832	1q41	IMPUT	A	G	0.730	0.270	0.999	0.998	0.716	0.284	0.999	0.997
1	222831372	rs2088514	1q41	IMPUT	A	G	0.824	0.176	0.998	0.995	0.819	0.182	0.998	0.993
1	222832295	rs2291834	1q41	IMPUT	C	T	0.730	0.270	0.998	0.996	0.716	0.284	0.998	0.995
1	222835222	rs17163384	1q41	IMPUT	A	C	0.933	0.068	0.999	0.994	0.927	0.073	0.999	0.994
1	222837594	rs2270707	1q41	GENOT	A	G	0.797	0.203	1	1	0.788	0.212	1	1
1	222839838	rs1053316	1q41	GENOT	G	A	0.905	0.095	1	1	0.897	0.103	1	1
1	222844840	rs3008633	1q41	IMPUT	C	T	0.918	0.082	0.974	0.860	0.907	0.093	0.974	0.877
1	222848029	rs1391558	1q41	IMPUT	T	C	0.827	0.174	0.976	0.916	0.822	0.179	0.973	0.911
9	21902354	rs756641	9p21	IMPUT	A	C	0.150	0.150	0.744	0.166	0.146	0.146	0.736	0.105
9	21905379	rs10811634	9p21	IMPUT	T	C	0.144	0.144	0.750	0.151	0.142	0.142	0.742	0.095
9	21909979	rs16938590	9p21	IMPUT	A	G	0.938	0.062	0.887	0.183	0.942	0.058	0.889	0.111
9	21920346	rs4977746	9p21	IMPUT	T	C	0.834	0.166	0.936	0.791	0.843	0.157	0.938	0.796
9	21923279	rs10811638	9p21	IMPUT	A	G	0.834	0.166	0.943	0.818	0.843	0.157	0.944	0.819
9	21925855	rs7852128	9p21	IMPUT	T	G	0.823	0.177	0.972	0.912	0.834	0.167	0.974	0.915
9	21927913	rs10965186	9p21	IMPUT	G	A	0.823	0.177	0.980	0.938	0.834	0.167	0.978	0.932
9	21929666	rs2518713	9p21	GENOT	A	G	0.824	0.176	1	1	0.833	0.167	1	1
9	21930147	rs7864029	9p21	GENOT	G	C	0.820	0.180	1	1	0.828	0.173	1	1
9	21931896	rs7869004	9p21	GENOT	G	T	0.820	0.180	1	1	0.828	0.173	1	1
9	21944317	rs4977750	9p21	IMPUT	A	C	0.865	0.135	0.952	0.821	0.890	0.111	0.962	0.832
9	21946322	rs2811717	9p21	IMPUT	T	C	0.869	0.131	0.974	0.909	0.896	0.104	0.985	0.933
9	21947957	rs2811720	9p21	GENOT	C	G	0.861	0.139	1	1	0.890	0.110	1	1
9	21948666	rs10965197	9p21	GENOT	C	T	0.604	0.396	1	1	0.632	0.368	1	1
9	21953137	rs10757260	9p21	IMPUT	A	G	0.596	0.405	0.994	0.989	0.645	0.355	0.993	0.986
9	21954953	rs10757261	9p21	GENOT	G	A	0.596	0.405	1	1	0.645	0.355	1	1
9	21955669	rs12335941	9p21	IMPUT	A	G	0.595	0.405	0.994	0.988	0.644	0.356	0.996	0.992
9	21958524	rs717326	9p21	GENOT	T	C	0.919	0.081	1	1	0.932	0.068	1	1
9	21961866	rs7041637	9p21	IMPUT	C	A	0.690	0.311	0.792	0.605	0.674	0.326	0.755	0.530
9	21966221	rs3731257	9p21	IMPUT	G	A	0.737	0.263	0.755	0.509	0.718	0.282	0.723	0.454
9	21970427	rs2518719	9p21	IMPUT	A	G	0.851	0.149	0.977	0.923	0.837	0.163	0.965	0.889
9	21973422	rs2811708	9p21	IMPUT	G	T	0.669	0.331	0.974	0.952	0.702	0.298	0.962	0.925
9	21974218	rs3731239	9p21	IMPUT	G	A	0.314	0.314	0.775	0.590	0.340	0.340	0.747	0.552
9	21983914	rs3731222	9p21	IMPUT	T	C	0.855	0.145	0.991	0.968	0.841	0.159	0.987	0.958
9	21986218	rs3731213	9p21	GENOT	C	T	0.979	0.021	1	1	0.991	0.009	1	1
9	21986847	rs3731211	9p21	IMPUT	A	T	0.687	0.313	0.991	0.982	0.710	0.290	0.988	0.974
9	21987584	rs3731204	9p21	IMPUT	T	C	0.855	0.145	0.993	0.977	0.841	0.159	0.989	0.965
9	21988896	rs3731201	9p21	GENOT	T	C	0.829	0.171	1	1	0.867	0.134	1	1
9	21990457	rs7036656	9p21	IMPUT	T	C	0.686	0.314	1.00	0.99	0.71	0.29	0.99	0.98
9	21991923	rs2811710	9p21	GENOT	C	T	0.598	0.402	1	1	0.646	0.355	1	1
9	21993964	rs2811711	9p21	GENOT	T	C	0.854	0.146	1	1	0.835	0.165	1	1
9	21997872	rs3218020	9p21	IMPUT	A	G	0.440	0.440	0.975	0.957	0.441	0.441	0.974	0.949
9	22000841	rs3218002	9p21	IMPUT	G	A	0.875	0.125	0.996	0.983	0.906	0.094	0.994	0.974
9	22003223	rs3217992	9p21	GENOT	T	C	0.481	0.481	1	1	0.474	0.474	1	1
9	22005330	rs3217986	9p21	GENOT	T	G	0.927	0.073	1	1	0.942	0.058	1	1
9	22006348	rs974336	9p21	IMPUT	C	T	0.875	0.125	0.997	0.986	0.906	0.094	0.994	0.976
9	22008026	rs2069422	9p21	IMPUT	T	G	0.872	0.128	0.994	0.979	0.904	0.096	0.993	0.970
9	22009698	rs2069418	9p21	IMPUT	C	G	0.657	0.343	0.992	0.985	0.606	0.394	0.991	0.985
9	22011477	rs575427	9p21	GENOT	A	G	0.920	0.081	1	1	0.894	0.106	1	1
9	22013411	rs10811640	9p21	IMPUT	T	G	0.534	0.467	0.995	0.992	0.513	0.487	0.993	0.988
9	22017836	rs643319	9p21	GENOT	C	A	0.620	0.380	1	1	0.591	0.409	1	1
9	22018781	rs7044859	9p21	GENOT	A	T	0.533	0.467	1	1	0.514	0.486	1	1
9	22019129	rs523096	9p21	GENOT	A	G	0.662	0.338	1	1	0.614	0.386	1	1
9	22019673	rs518394	9p21	GENOT	G	C	0.664	0.336	1	1	0.615	0.386	1	1

9	22019732	rs10757264	9p21	GENOT	G	A	0.580	0.420	1	1	0.561	0.439	1	1
9	22023795	rs10965212	9p21	GENOT	A	T	0.569	0.431	1	1	0.544	0.456	1	1
9	22024351	rs496892	9p21	GENOT	C	T	0.378	0.378	1	1	0.406	0.406	1	1
9	22025493	rs10738604	9p21	IMPUT	A	G	0.460	0.460	0.991	0.980	0.458	0.458	0.993	0.982
9	22026834	rs1591136	9p21	IMPUT	C	G	0.563	0.437	0.985	0.972	0.537	0.463	0.985	0.970
9	22027551	rs598664	9p21	IMPUT	T	C	0.875	0.125	0.995	0.983	0.906	0.094	0.995	0.979
9	22028801	rs7049105	9p21	GENOT	G	A	0.570	0.430	1	1	0.544	0.456	1	1
9	22029445	rs10965215	9p21	GENOT	A	G	0.563	0.438	1	1	0.537	0.463	1	1
9	22029547	rs564398	9p21	GENOT	T	C	0.697	0.303	1	1	0.642	0.359	1	1
9	22030438	rs662463	9p21	GENOT	G	A	0.881	0.119	1	1	0.908	0.092	1	1
9	22031005	rs7865618	9p21	GENOT	A	G	0.691	0.309	1	1	0.635	0.365	1	1
9	22032119	rs10115049	9p21	IMPUT	G	A	0.568	0.432	0.997	0.995	0.543	0.457	0.998	0.996
9	22033366	rs2157719	9p21	IMPUT	T	C	0.691	0.309	0.997	0.993	0.634	0.366	0.996	0.992
9	22036112	rs1008878	9p21	IMPUT	T	G	0.691	0.309	0.996	0.990	0.634	0.366	0.994	0.988
9	22039426	rs12376000	9p21	IMPUT	C	T	0.917	0.083	0.991	0.951	0.894	0.106	0.990	0.952
9	22041998	rs17694493	9p21	GENOT	C	G	0.858	0.142	1	1	0.874	0.126	1	1
9	22042086	rs12352425	9p21	GENOT	G	A	0.925	0.075	1	1	0.940	0.060	1	1
9	22043926	rs1412829	9p21	IMPUT	A	G	0.692	0.308	0.985	0.972	0.634	0.367	0.989	0.978
9	22045317	rs1360589	9p21	IMPUT	T	C	0.691	0.309	0.988	0.979	0.636	0.364	0.993	0.987
9	22048683	rs7028570	9p21	IMPUT	A	G	0.566	0.434	0.983	0.976	0.545	0.455	0.989	0.983
9	22053895	rs17756311	9p21	GENOT	G	A	0.895	0.105	1	1	0.919	0.081	1	1
9	22054356	rs17694572	9p21	GENOT	G	A	0.896	0.104	1	1	0.917	0.084	1	1
9	22056499	rs10120688	9p21	GENOT	A	G	0.574	0.426	1	1	0.554	0.446	1	1
9	22061614	rs1537378	9p21	IMPUT	G	A	0.708	0.292	0.992	0.984	0.657	0.343	0.993	0.987
9	22062134	rs1011970	9p21	IMPUT	G	T	0.817	0.184	0.983	0.955	0.856	0.144	0.981	0.946
9	22064465	rs8181047	9p21	GENOT	G	A	0.785	0.215	1	1	0.750	0.250	1	1
9	22065002	rs10811647	9p21	IMPUT	G	C	0.515	0.485	0.987	0.979	0.510	0.490	0.984	0.975
9	22067276	rs10965224	9p21	GENOT	A	T	0.707	0.293	1	1	0.657	0.343	1	1
9	22069144	rs16905599	9p21	GENOT	G	A	0.931	0.069	1	1	0.943	0.057	1	1
9	22072301	rs9632884	9p21	GENOT	C	G	0.623	0.377	1	1	0.600	0.400	1	1
9	22072719	rs10757270	9p21	GENOT	G	A	0.516	0.484	1	1	0.514	0.486	1	1
9	22073334	rs16923583	9p21	GENOT	T	A	0.977	0.023	1	1	0.989	0.011	1	1
9	22077543	rs1412832	9p21	IMPUT	T	C	0.781	0.220	0.961	0.912	0.750	0.250	0.963	0.926
9	22081850	rs6475606	9p21	GENOT	T	C	0.609	0.391	1	1	0.595	0.405	1	1
9	22082340	rs1547704	9p21	GENOT	G	A	0.982	0.018	1	1	0.987	0.013	1	1
9	22082380	rs10965228	9p21	GENOT	A	G	0.911	0.089	1	1	0.902	0.098	1	1
9	22083404	rs1333040	9p21	IMPUT	T	C	0.687	0.313	0.984	0.969	0.687	0.314	0.987	0.974
9	22087473	rs7857345	9p21	IMPUT	C	T	0.798	0.202	0.919	0.808	0.767	0.233	0.920	0.821
9	22088260	rs10757272	9p21	GENOT	T	C	0.591	0.409	1	1	0.575	0.425	1	1
9	22098574	rs4977574	9p21	GENOT	G	A	0.593	0.407	1	1	0.575	0.425	1	1
9	22098619	rs2891168	9p21	GENOT	G	A	0.592	0.408	1	1	0.575	0.425	1	1
9	22099568	rs1537371	9p21	IMPUT	A	C	0.604	0.396	0.997	0.995	0.582	0.419	0.995	0.992
9	22102165	rs7859727	9p21	IMPUT	T	C	0.594	0.406	0.997	0.994	0.576	0.424	0.996	0.993
9	22103813	rs1333042	9p21	GENOT	G	A	0.629	0.371	1	1	0.603	0.397	1	1
9	22105927	rs7859362	9p21	IMPUT	C	T	0.610	0.390	0.980	0.963	0.589	0.412	0.978	0.960
9	22106731	rs1333043	9p21	IMPUT	A	T	0.610	0.390	0.979	0.959	0.588	0.412	0.977	0.957
9	22110131	rs1412834	9p21	IMPUT	C	T	0.609	0.391	0.976	0.953	0.588	0.412	0.974	0.950
9	22112241	rs7341786	9p21	IMPUT	C	A	0.615	0.385	0.961	0.927	0.595	0.405	0.957	0.919
9	22114469	rs10733376	9p21	IMPUT	C	G	0.611	0.389	0.969	0.944	0.590	0.410	0.965	0.936
9	22115026	rs2383206	9p21	IMPUT	G	A	0.611	0.389	0.970	0.944	0.590	0.410	0.965	0.936
9	22115959	rs2383207	9p21	IMPUT	G	A	0.611	0.389	0.970	0.945	0.591	0.410	0.966	0.937
9	22119195	rs1333045	9p21	IMPUT	C	T	0.586	0.414	0.962	0.931	0.558	0.442	0.965	0.937
9	22123766	rs10738610	9p21	IMPUT	C	A	0.599	0.401	0.991	0.984	0.582	0.418	0.993	0.985
9	22124477	rs10757278	9p21	IMPUT	G	A	0.566	0.435	0.982	0.969	0.550	0.451	0.987	0.975
9	22125347	rs1333048	9p21	GENOT	C	A	0.597	0.403	1	1	0.579	0.421	1	1
9	22125503	rs1333049	9p21	GENOT	C	G	0.562	0.438	1	1	0.548	0.452	1	1
9	22127613	rs10757281	9p21	IMPUT	C	T	0.864	0.136	0.802	0.310	0.849	0.151	0.825	0.419
9	22128709	rs12347779	9p21	GENOT	C	G	0.946	0.054	1	1	0.922	0.078	1	1
9	22130065	rs10965243	9p21	IMPUT	A	G	0.921	0.079	0.995	0.967	0.894	0.106	0.998	0.989
9	22130515	rs10965245	9p21	GENOT	G	A	0.921	0.079	1	1	0.893	0.107	1	1
9	22131825	rs2891169	9p21	GENOT	A	G	0.572	0.428	1	1	0.553	0.448	1	1
9	22132076	rs2383208	9p21	GENOT	A	G	0.798	0.202	1	1	0.799	0.201	1	1
9	22133251	rs7045889	9p21	GENOT	G	A	0.334	0.334	1	1	0.356	0.356	1	1
9	22133716	rs10811659	9p21	GENOT	T	C	0.756	0.244	1	1	0.776	0.224	1	1
9	22133984	rs10757282	9p21	GENOT	T	C	0.529	0.471	1	1	0.522	0.479	1	1
9	22134094	rs10811661	9p21	GENOT	T	C	0.803	0.197	1	1	0.807	0.193	1	1
9	22134172	rs10757283	9p21	GENOT	C	T	0.527	0.473	1	1	0.525	0.475	1	1
9	22136489	rs1333051	9p21	GENOT	A	T	0.844	0.156	1	1	0.842	0.158	1	1
9	22137685	rs7018475	9p21	GENOT	T	G	0.700	0.300	1	1	0.708	0.293	1	1
9	22138105	rs11791416	9p21	GENOT	G	A	0.297	0.297	1	1	0.275	0.275	1	1
9	22138762	rs4977761	9p21	GENOT	T	C	0.283	0.283	1	1	0.305	0.305	1	1

9	22140224	rs2065501	9p21	IMPUT	C	A	0.565	0.435	0.559	0.213	0.594	0.406	0.550	0.190
9	22141875	rs4977577	9p21	IMPUT	C	T	0.273	0.273	0.704	0.418	0.292	0.292	0.708	0.452
9	22143293	rs7849199	9p21	IMPUT	T	A	0.703	0.297	0.916	0.804	0.714	0.287	0.946	0.873
9	22145694	rs2065500	9p21	IMPUT	A	G	0.781	0.219	0.946	0.863	0.775	0.225	0.961	0.902
9	22147715	rs7022662	9p21	GENOT	C	G	0.778	0.222	1	1	0.775	0.226	1	1
9	22148055	rs12341394	9p21	GENOT	C	T	0.701	0.299	1	1	0.715	0.285	1	1
9	22150261	rs7856219	9p21	IMPUT	T	C	0.696	0.304	0.989	0.977	0.707	0.293	0.979	0.954
9	22151465	rs10965256	9p21	GENOT	G	A	0.926	0.074	1	1	0.930	0.070	1	1
9	22153360	rs7853123	9p21	IMPUT	A	G	0.448	0.448	0.891	0.821	0.459	0.459	0.887	0.807
9	22155709	rs944802	9p21	IMPUT	C	T	0.789	0.211	0.958	0.907	0.777	0.223	0.956	0.906
9	22157360	rs7028213	9p21	IMPUT	T	G	0.695	0.305	0.972	0.939	0.709	0.291	0.967	0.928
9	22158168	rs12238587	9p21	IMPUT	T	A	0.780	0.220	0.986	0.968	0.771	0.229	0.987	0.969
9	22159416	rs10757288	9p21	IMPUT	C	T	0.404	0.404	0.955	0.928	0.427	0.427	0.950	0.919
9	22161212	rs7864275	9p21	GENOT	C	T	0.626	0.374	1	1	0.660	0.340	1	1
9	22161494	rs10965266	9p21	GENOT	T	G	0.782	0.218	1	1	0.774	0.226	1	1
9	22161828	rs10965267	9p21	GENOT	T	G	0.917	0.083	1	1	0.941	0.059	1	1
9	22164991	rs10811668	9p21	GENOT	C	A	0.726	0.274	1	1	0.751	0.249	1	1
9	22166769	rs2779748	9p21	IMPUT	T	C	0.379	0.379	0.877	0.791	0.411	0.411	0.864	0.782
9	22168128	rs7863846	9p21	GENOT	C	T	0.708	0.292	1	1	0.723	0.277	1	1
9	22168464	rs828580	9p21	GENOT	A	T	0.926	0.074	1	1	0.949	0.051	1	1
9	22169700	rs1537377	9p21	GENOT	T	C	0.635	0.365	1	1	0.673	0.327	1	1
9	22170983	rs954399	9p21	GENOT	C	A	0.708	0.292	1	1	0.728	0.272	1	1
9	22172259	rs828582	9p21	IMPUT	T	A	0.615	0.385	0.953	0.909	0.655	0.345	0.955	0.915
9	22174712	rs10965278	9p21	GENOT	G	A	0.728	0.272	1	1	0.733	0.267	1	1
9	22175188	rs10965279	9p21	GENOT	G	C	0.931	0.069	1	1	0.932	0.068	1	1
9	22176961	rs10757292	9p21	IMPUT	C	T	0.963	0.037	0.937	0.243	0.969	0.031	0.944	0.186
9	22183781	rs866666	9p21	IMPUT	C	T	0.340	0.340	0.639	0.335	0.368	0.368	0.629	0.337
9	22184997	rs2767409	9p21	IMPUT	G	A	0.627	0.373	0.649	0.369	0.653	0.347	0.646	0.342
9	22187074	rs1095904	9p21	IMPUT	G	T	0.656	0.344	0.629	0.317	0.680	0.320	0.632	0.298
9	22191189	rs828576	9p21	IMPUT	T	C	0.341	0.341	0.631	0.315	0.367	0.367	0.622	0.321
9	22195820	rs2219849	9p21	IMPUT	T	C	0.531	0.469	0.604	0.309	0.539	0.461	0.595	0.293
9	22196863	rs1751449	9p21	IMPUT	A	G	0.599	0.401	0.635	0.343	0.620	0.380	0.634	0.331
9	22198149	rs12375458	9p21	IMPUT	G	A	0.898	0.102	0.812	0.115	0.904	0.096	0.819	0.085
10	44682973	rs1482472	10q11	IMPUT	T	C	0.624	0.376	0.858	0.721	0.624	0.376	0.831	0.677
10	44686664	rs12415866	10q11	IMPUT	A	G	0.867	0.133	0.959	0.857	0.895	0.105	0.958	0.804
10	44688587	rs7917089	10q11	IMPUT	G	A	0.868	0.132	0.963	0.881	0.899	0.101	0.960	0.819
10	44691241	rs1623851	10q11	GENOT	A	G	0.814	0.186	1	1	0.836	0.165	1	1
10	44691633	rs1627329	10q11	GENOT	T	C	0.813	0.187	1	1	0.836	0.165	1	1
10	44693544	rs10508883	10q11	GENOT	A	C	0.976	0.024	1	1	0.981	0.019	1	1
10	44693742	rs7900182	10q11	GENOT	T	G	0.829	0.171	1	1	0.807	0.193	1	1
10	44694201	rs11597731	10q11	GENOT	C	T	0.822	0.178	1	1	0.801	0.200	1	1
10	44695308	rs7902040	10q11	GENOT	A	G	0.889	0.111	1	1	0.917	0.084	1	1
10	44695862	rs11238911	10q11	GENOT	G	A	0.891	0.110	1	1	0.917	0.083	1	1
10	44695973	rs11238913	10q11	GENOT	T	C	0.667	0.333	1	1	0.661	0.339	1	1
10	44696034	rs2802477	10q11	IMPUT	G	A	0.418	0.418	0.781	0.665	0.419	0.419	0.775	0.660
10	44696352	rs11594522	10q11	GENOT	G	A	0.833	0.168	1	1	0.808	0.192	1	1
10	44698075	rs2054620	10q11	IMPUT	T	C	0.667	0.333	0.994	0.987	0.661	0.339	0.995	0.990
10	44699910	rs11238921	10q11	IMPUT	G	T	0.667	0.333	0.991	0.980	0.662	0.339	0.992	0.983
10	44702681	rs768676	10q11	GENOT	T	A	0.938	0.062	1	1	0.941	0.060	1	1
10	44705969	rs3865770	10q11	IMPUT	G	A	0.832	0.168	0.990	0.960	0.807	0.193	0.991	0.972
10	44707598	rs1482473	10q11	IMPUT	G	C	0.845	0.155	0.990	0.963	0.861	0.139	0.992	0.968
10	44709171	rs3851257	10q11	IMPUT	G	T	0.668	0.333	0.983	0.957	0.662	0.339	0.985	0.967
10	44712128	rs12573558	10q11	IMPUT	C	A	0.818	0.182	0.978	0.927	0.797	0.203	0.982	0.948
10	44714402	rs11238935	10q11	IMPUT	C	T	0.891	0.109	0.992	0.961	0.918	0.082	0.994	0.962
10	44716469	rs2209067	10q11	IMPUT	G	A	0.818	0.183	0.977	0.926	0.796	0.204	0.982	0.947
10	44729958	rs1704219	10q11	GENOT	G	C	0.682	0.318	1	1	0.676	0.324	1	1
10	44730995	rs7907961	10q11	IMPUT	T	C	0.791	0.209	0.994	0.985	0.757	0.243	0.993	0.984
10	44732825	rs1746043	10q11	IMPUT	T	C	0.679	0.321	0.990	0.983	0.677	0.323	0.987	0.980
10	44734995	rs647419	10q11	IMPUT	G	A	0.639	0.361	0.990	0.979	0.638	0.362	0.983	0.971
10	44737036	rs88796	10q11	GENOT	T	C	0.722	0.278	1	1	0.735	0.266	1	1
10	44737246	rs617019	10q11	GENOT	G	A	0.886	0.114	1	1	0.921	0.080	1	1
10	44737433	rs17155733	10q11	GENOT	A	G	0.837	0.163	1	1	0.817	0.183	1	1
10	44738688	rs583489	10q11	GENOT	C	G	0.876	0.124	1	1	0.913	0.087	1	1
10	44739594	rs676966	10q11	GENOT	C	T	0.884	0.116	1	1	0.919	0.081	1	1
10	44741256	rs494207	10q11	IMPUT	G	A	0.876	0.124	0.999	0.994	0.913	0.087	0.998	0.987
10	44746395	rs541483	10q11	IMPUT	A	G	0.826	0.174	1.000	0.999	0.864	0.136	1.000	0.998
10	44747059	rs535176	10q11	GENOT	C	T	0.826	0.174	1	1	0.864	0.136	1	1
10	44749211	rs622472	10q11	GENOT	A	C	0.826	0.174	1	1	0.864	0.136	1	1
10	44749708	rs513391	10q11	GENOT	A	C	0.826	0.174	1	1	0.864	0.136	1	1
10	44749854	rs11238956	10q11	GENOT	C	T	0.339	0.339	1	1	0.329	0.329	1	1
10	44751910	rs687175	10q11	GENOT	T	C	0.827	0.173	1	1	0.867	0.133	1	1

10	44752078	rs559580	10q11	GENOT	T	C	0.829	0.171	1	1	0.872	0.128	1	1
10	44752118	rs559469	10q11	GENOT	T	C	0.826	0.174	1	1	0.867	0.133	1	1
10	44752268	rs2437935	10q11	GENOT	A	G	0.614	0.386	1	1	0.624	0.376	1	1
10	44752330	rs535949	10q11	GENOT	G	T	0.828	0.172	1	1	0.867	0.133	1	1
10	44752976	rs671765	10q11	GENOT	A	G	0.828	0.172	1	1	0.867	0.133	1	1
10	44753867	rs501120	10q11	IMPUT	T	C	0.828	0.173	0.999	0.997	0.867	0.133	0.999	0.996
10	44755104	rs579058	10q11	IMPUT	A	G	0.828	0.173	0.999	0.996	0.867	0.133	0.999	0.995
10	44756894	rs604674	10q11	IMPUT	G	T	0.828	0.172	0.995	0.985	0.867	0.133	0.997	0.986
10	44758197	rs487465	10q11	IMPUT	A	C	0.828	0.172	0.995	0.984	0.867	0.133	0.997	0.986
10	44760887	rs475926	10q11	IMPUT	T	G	0.678	0.322	0.984	0.970	0.687	0.313	0.986	0.972
10	44773984	rs1632484	10q11	GENOT	C	T	0.833	0.168	1	1	0.872	0.128	1	1
10	44775824	rs1746048	10q11	GENOT	C	T	0.830	0.170	1	1	0.868	0.132	1	1
10	44776310	rs1746049	10q11	GENOT	C	T	0.832	0.168	1	1	0.869	0.131	1	1
10	44778546	rs1746052	10q11	GENOT	A	C	0.838	0.162	1	1	0.871	0.129	1	1
10	44786364	rs800314	10q11	IMPUT	A	G	0.915	0.085	0.994	0.970	0.932	0.068	0.990	0.940
10	44791433	rs11598523	10q11	IMPUT	T	A	0.841	0.160	0.995	0.983	0.812	0.188	0.992	0.979
10	44793299	rs2505734	10q11	GENOT	T	C	0.631	0.369	1	1	0.655	0.345	1	1
10	44797087	rs754713	10q11	GENOT	C	T	0.717	0.283	1	1	0.726	0.274	1	1
10	44798482	rs800320	10q11	GENOT	C	T	0.956	0.044	1	1	0.967	0.033	1	1
10	44801673	rs800323	10q11	GENOT	A	G	0.717	0.284	1	1	0.724	0.276	1	1
10	44803925	rs2437934	10q11	GENOT	C	G	0.632	0.368	1	1	0.654	0.346	1	1
10	44810205	rs11238983	10q11	GENOT	A	G	0.165	0.165	1	1	0.135	0.135	1	1
10	44813738	rs2146807	10q11	GENOT	C	T	0.164	0.164	1	1	0.139	0.139	1	1
10	44813777	rs2146808	10q11	GENOT	A	C	0.959	0.041	1	1	0.970	0.030	1	1
10	44814336	rs7082209	10q11	GENOT	G	A	0.168	0.168	1	1	0.134	0.134	1	1
10	44815048	rs800310	10q11	GENOT	A	T	0.232	0.232	1	1	0.179	0.179	1	1
10	44815709	rs2505735	10q11	GENOT	C	A	0.790	0.210	1	1	0.835	0.165	1	1
10	44817419	rs977754	10q11	GENOT	T	G	0.803	0.197	1	1	0.847	0.153	1	1
10	44818563	rs812889	10q11	GENOT	C	G	0.912	0.088	1	1	0.936	0.064	1	1
10	44820157	rs2476351	10q11	IMPUT	T	C	0.807	0.194	0.989	0.976	0.853	0.147	0.992	0.977
10	44821220	rs11238987	10q11	GENOT	G	A	0.967	0.033	1	1	0.968	0.032	1	1
10	44821387	rs10508884	10q11	GENOT	C	T	0.731	0.269	1	1	0.711	0.289	1	1
10	44821772	rs1111259	10q11	GENOT	C	T	0.887	0.113	1	1	0.915	0.085	1	1
10	44823136	rs2505741	10q11	GENOT	A	G	0.525	0.475	1	1	0.553	0.448	1	1
10	44826376	rs2028100	10q11	GENOT	G	A	0.754	0.246	1	1	0.728	0.272	1	1
10	44827164	rs1836982	10q11	GENOT	C	G	0.787	0.214	1	1	0.841	0.159	1	1
10	44828234	rs982097	10q11	IMPUT	A	G	0.537	0.463	0.998	0.997	0.567	0.433	0.998	0.998
10	44830727	rs7918046	10q11	GENOT	C	T	0.705	0.296	1	1	0.719	0.281	1	1
10	44831379	rs11815919	10q11	GENOT	C	T	0.817	0.183	1	1	0.805	0.195	1	1
10	44832884	rs928565	10q11	GENOT	A	G	0.490	0.490	1	1	0.529	0.471	1	1
10	44833031	rs11599561	10q11	GENOT	T	C	0.892	0.108	1	1	0.889	0.111	1	1
10	44833894	rs266080	10q11	GENOT	A	G	0.978	0.022	1	1	0.983	0.017	1	1
10	44835963	rs1144482	10q11	GENOT	T	C	0.477	0.477	1	1	0.516	0.484	1	1
10	44837267	rs1360724	10q11	IMPUT	A	G	0.682	0.318	0.999	0.999	0.698	0.303	0.999	0.998
10	44838019	rs10900025	10q11	GENOT	A	G	0.593	0.407	1	1	0.603	0.398	1	1
10	44838464	rs7069891	10q11	GENOT	C	T	0.891	0.109	1	1	0.889	0.111	1	1
10	44838530	rs77839	10q11	GENOT	A	G	0.571	0.429	1	1	0.586	0.414	1	1
10	44839865	rs17390084	10q11	GENOT	A	G	0.042	0.042	1	1	0.034	0.034	1	1
10	44842048	rs10793536	10q11	IMPUT	G	C	0.562	0.438	0.996	0.993	0.582	0.418	0.993	0.989
10	44844381	rs266076	10q11	IMPUT	A	G	0.560	0.440	0.997	0.995	0.580	0.420	0.996	0.993
10	44848921	rs1144480	10q11	IMPUT	A	T	0.462	0.462	0.979	0.965	0.502	0.498	0.988	0.979
10	44850424	rs266109	10q11	GENOT	A	G	0.878	0.122	1	1	0.900	0.100	1	1
10	44851737	rs1144477	10q11	GENOT	T	C	0.478	0.478	1	1	0.512	0.488	1	1
10	44855663	rs266105	10q11	GENOT	G	A	0.862	0.139	1	1	0.889	0.111	1	1
10	44855740	rs11595588	10q11	GENOT	T	C	0.603	0.397	1	1	0.612	0.388	1	1
10	44855927	rs17391002	10q11	GENOT	A	G	0.801	0.199	1	1	0.767	0.233	1	1
10	44856370	rs266103	10q11	GENOT	C	T	0.855	0.145	1	1	0.890	0.111	1	1
10	44858840	rs185545	10q11	IMPUT	G	C	0.793	0.207	0.894	0.752	0.799	0.201	0.868	0.706
10	44861220	rs7918568	10q11	GENOT	C	T	0.829	0.171	1	1	0.814	0.186	1	1
10	44863434	rs7915848	10q11	GENOT	T	C	0.828	0.172	1	1	0.816	0.184	1	1
10	44864300	rs266094	10q11	GENOT	C	T	0.854	0.146	1	1	0.875	0.125	1	1
10	44866208	rs266093	10q11	IMPUT	G	C	0.639	0.361	0.997	0.994	0.658	0.342	0.993	0.988
10	44867146	rs1029153	10q11	GENOT	A	G	0.725	0.275	1	1	0.721	0.279	1	1
10	44868257	rs1801157	10q11	GENOT	T	C	0.246	0.246	1	1	0.215	0.215	1	1
10	44869427	rs266089	10q11	GENOT	G	A	0.847	0.153	1	1	0.859	0.141	1	1
10	44870015	rs266088	10q11	GENOT	C	T	0.853	0.147	1	1	0.814	0.186	1	1
10	44871062	rs266087	10q11	IMPUT	A	G	0.399	0.399	0.934	0.875	0.403	0.403	0.951	0.904
10	44871548	rs2297630	10q11	IMPUT	G	A	0.731	0.269	0.918	0.812	0.730	0.270	0.936	0.848
10	44875166	rs2839690	10q11	IMPUT	A	G	0.833	0.167	0.927	0.759	0.824	0.176	0.922	0.750
10	44878713	rs3780891	10q11	IMPUT	G	A	0.915	0.085	0.925	0.605	0.890	0.110	0.896	0.581

Al1 and Al2, referenced allele; BP, genomic position in base pair; CHR, chromosome; GENOT, genotyped; IMPUT, imputed; MAF, minor allele frequency; Quality, the average posterior probability for the most likely genotype; Rsq, MACH quality metric (R2); SNP, single nucleotide polymorphism.
SNPs with low imputation quality (R2<0.3) are highlighted in grey.

Chromosome positions from Genome Reference Consortium human build 37 (GRCh37).
