# Supplementary material for: Analysis of Genomic Regions Associated With Coronary Artery Disease Reveals Continent-Specific Single Nucleotide Polymorphisms in North African Populations
Source: J Epidemiol. 2016 May 5;26(5):264–71. doi: 10.2188/jea.JE20150034 (PMC4848325; doi:10.2188/jea.JE20150034)
Supplement: eTable 2. [file je-26-264-s002.pdf]

eTable 2. Case-control allelic frequencies and association parameters adjusted for gender in the genotype and imputed case-control samples

				Morocco							Tunisia							ATVB							Regicor						
CHR	SNP	BP	Region	A1	F_A	F_U	OR	L95	U95	P	A1	F_A	F_U	OR	L95	U95	P	A1	F_A	F_U	OR	L95	U95	P	A1	F_A	F_U	OR	L95	U95	P
1	rs4970833	109804646	1p13	A	0.493	0.418	1.268	0.792	2.032	0.330	A	0.533	0.493	1.426	0.741	2.744	0.288														
1	rs653635	109806313	1p13	G	0.069	0.214	0.257	0.110	0.598	0.003	G	0.120	0.092	0.884	0.323	2.417	0.841														
1	rs6657811	109807283	1p13	T	0.142	0.085	1.828	0.746	4.483	0.208	T	0.076	0.112	1.050	0.345	3.196	0.912	T	0.084	0.096	0.868	0.735	1.025	0.096	T	0.107	0.085	1.292	0.879	1.898	0.180
1	rs608196	109808117	1p13	A	0.028	0.082	0.317	0.089	1.129	0.055	A	0.098	0.033	2.188	0.585	8.188	0.243														
1	rs17035630	109810981	1p13	A	0.070	0.122	0.589	0.250	1.390	0.253	A	0.098	0.072	1.031	0.386	2.752	0.981														
1	rs17035665	109813719	1p13	T	0.220	0.167	1.334	0.695	2.563	0.386	T	0.174	0.224	0.866	0.401	1.873	0.718														
1	rs4970834	109814880	1p13	T	0.215	0.229	0.928	0.519	1.659	0.777	T	0.087	0.197	0.314	0.118	0.835	0.020	T	0.121	0.143	0.831	0.721	0.956	0.013	T	0.161	0.136	1.213	0.896	1.643	0.198
1	rs611917	109815252	1p13	C	0.236	0.245	0.974	0.540	1.755	0.977	C	0.207	0.243	0.772	0.353	1.690	0.526	G	0.217	0.232	0.912	0.809	1.027	0.130	G	0.277	0.252	1.160	0.888	1.516	0.256
1	rs12740374	109817590	1p13	T	0.190	0.177	1.088	0.566	2.091	0.788	T	0.120	0.224	0.541	0.228	1.285	0.171	T	0.170	0.191	0.870	0.769	0.985	0.029	T	0.203	0.185	1.129	0.855	1.491	0.359
1	rs629301	109818306	1p13	C	0.201	0.194	1.038	0.563	1.917	0.988	C	0.141	0.270	0.475	0.206	1.095	0.063	G	0.170	0.191	0.870	0.769	0.985	0.029	G	0.203	0.185	1.129	0.855	1.491	0.359
1	rs646776	109818530	1p13	G	0.201	0.194	1.038	0.563	1.917	0.988	G	0.141	0.270	0.475	0.206	1.095	0.063	C	0.170	0.191	0.870	0.769	0.985	0.029	C	0.203	0.185	1.129	0.855	1.491	0.359
1	rs17035949	109820919	1p13	G	0.118	0.174	0.585	0.274	1.246	0.148	G	0.065	0.105	0.601	0.192	1.882	0.364	G	0.053	0.052	1.020	0.826	1.261	0.863	G	0.053	0.054	1.002	0.612	1.640	0.997
1	rs583104	109821307	1p13	C	0.319	0.378	0.769	0.457	1.293	0.306	C	0.174	0.342	0.358	0.156	0.821	0.010	G	0.184	0.203	0.885	0.784	0.999	0.055	G	0.224	0.196	1.192	0.909	1.563	0.181
1	rs602633	109821511	1p13	A	0.319	0.357	0.838	0.496	1.415	0.515	A	0.163	0.322	0.354	0.153	0.819	0.014	T	0.180	0.201	0.870	0.771	0.983	0.023	T	0.223	0.191	1.213	0.925	1.591	0.151
1	rs599839	109822166	1p13	G	0.319	0.367	0.805	0.480	1.351	0.403	G	0.174	0.336	0.365	0.159	0.837	0.011	G	0.184	0.202	0.892	0.790	1.007	0.073	G	0.223	0.197	1.170	0.893	1.534	0.221
1	rs14000	109822509	1p13	C	0.083	0.153	0.483	0.209	1.117	0.049	C	0.120	0.059	1.214	0.405	3.645	0.733	C	0.120	0.100	1.227	1.052	1.432	0.013	C	0.123	0.114	1.098	0.771	1.564	0.609
1	rs672569	109827253	1p13	A	0.188	0.255	0.710	0.398	1.266	0.265	A	0.141	0.243	0.382	0.159	0.918	0.028	A	0.103	0.114	0.897	0.768	1.046	0.166	A	0.124	0.110	1.152	0.814	1.630	0.390
1	rs655246	109832283	1p13	A	0.493	0.490	1.034	0.621	1.724	0.897	A	0.500	0.513	0.874	0.457	1.670	0.676	A	0.354	0.365	0.949	0.853	1.056	0.313	A	0.369	0.382	0.941	0.730	1.211	0.620
1	rs17584208	109833187	1p13	A	0.049	0.051	0.974	0.288	3.294	0.979	A	0.033	0.066	0.357	0.080	1.585	0.151	A	0.044	0.043	1.014	0.802	1.281	0.921	A	0.058	0.046	1.311	0.787	2.185	0.285
1	rs17645031	109834938	1p13	T	0.049	0.051	0.974	0.288	3.294	0.979	T	0.033	0.066	0.357	0.080	1.585	0.151	T	0.044	0.043	1.014	0.802	1.281	0.921	T	0.058	0.046	1.311	0.787	2.185	0.285
1	rs17645143	109835757	1p13	C	0.236	0.235	1.022	0.559	1.869	0.885	C	0.304	0.224	1.529	0.747	3.131	0.233	C	0.228	0.218	1.067	0.946	1.204	0.300	C	0.207	0.233	0.842	0.636	1.114	0.216
1	rs629001	109838918	1p13	C	0.097	0.163	0.532	0.236	1.200	0.140	C	0.109	0.171	1.054	0.410	2.709	0.895	C	0.030	0.045	0.653	0.506	0.843	0.002	C	0.048	0.041	1.170	0.695	1.972	0.566
1	rs3850615	109839738	1p13	A	0.104	0.082	1.307	0.524	3.256	0.641	A	0.054	0.099	0.744	0.230	2.411	0.648	A	0.041	0.031	1.343	1.036	1.740	0.023	A	0.039	0.052	0.748	0.443	1.262	0.260
1	rs600806	109840629	1p13	C	0.264	0.250	1.081	0.570	2.052	0.831	C	0.228	0.296	0.845	0.421	1.697	0.645	G	0.224	0.213	1.067	0.951	1.197	0.280	G	0.257	0.222	1.217	0.933	1.589	0.147
1	rs680434	109842271	1p13	G	0.285	0.255	1.193	0.649	2.194	0.553	G	0.261	0.283	0.944	0.473	1.885	0.871	C	0.255	0.238	1.093	0.979	1.221	0.114	C	0.277	0.252	1.141	0.885	1.471	0.313
1	rs11583969	109843775	1p13	T	0.063	0.092	0.599	0.217	1.656	0.291	T	0.022	0.039	0.335	0.056	2.001	0.236	T	0.054	0.044	1.229	0.988	1.529	0.068	T	0.081	0.049	1.672	1.057	2.646	0.029
1	rs652651	109844532	1p13	T	0.285	0.245	1.269	0.688	2.339	0.445	T	0.261	0.263	0.924	0.454	1.879	0.861	A	0.259	0.238	1.117	1.000	1.247	0.050	A	0.276	0.260	1.087	0.842	1.403	0.511
1	rs407102	109846278	1p13	G	0.243	0.235	1.077	0.570	2.032	0.809	G	0.261	0.290	0.820	0.407	1.652	0.560	C	0.252	0.232	1.112	0.995	1.242	0.059	C	0.266	0.251	1.085	0.840	1.401	0.536
1	rs592107	109848726	1p13	T	0.257	0.214	1.314	0.691	2.499	0.371	T	0.261	0.296	0.811	0.405	1.625	0.502	A	0.252	0.232	1.112	0.995	1.242	0.059	A	0.266	0.251	1.085	0.840	1.401	0.536
1	rs444387	109851127	1p13	A	0.304	0.260	1.284	0.691	2.388	0.431	A	0.272	0.329	0.743	0.370	1.493	0.396	A	0.253	0.234	1.104	0.989	1.234	0.080	A	0.266	0.256	1.058	0.820	1.365	0.675
1	rs464218	109856306	1p13	T	0.493	0.439	1.263	0.767	2.080	0.374	C	0.500	0.513	0.783	0.408	1.502	0.461	G	0.427	0.413	1.062	0.964	1.170	0.202	G	0.452	0.459	0.969	0.770	1.218	0.781
1	rs17585355	109857815	1p13	C	0.310	0.255	1.448	0.719	2.919	0.303	C	0.304	0.224	1.926	0.915	4.056	0.081	C	0.034	0.026	1.362	1.024	1.813	0.032	C	0.031	0.039	0.786	0.440	1.403	0.410
1	rs370088	109858119	1p13	T	0.264	0.214	1.348	0.718	2.531	0.354	T	0.261	0.296	0.811	0.405	1.625	0.502	T	0.252	0.234	1.103	0.987	1.232	0.087	T	0.266	0.256	1.058	0.820	1.365	0.675
1	rs3853500	109864269	1p13	A	0.292	0.255	1.236	0.671	2.277	0.460	A	0.272	0.329	0.743	0.370	1.493	0.396	T	0.251	0.232	1.111	0.994	1.242	0.061	T	0.265	0.251	1.075	0.832	1.389	0.566
1	rs3768496	109866569	1p13	A	0.257	0.214	1.314	0.691	2.499	0.371	A	0.261	0.296	0.811	0.405	1.625	0.502	T	0.258	0.236	1.121	1.004	1.252	0.042	T	0.273	0.259	1.077	0.833	1.392	0.583
1	rs10745352	109871787	1p13	T	0.292	0.255	1.236	0.671	2.277	0.460	T	0.272	0.329	0.743	0.370	1.493	0.396	T	0.258	0.236	1.121	1.004	1.252	0.042	T	0.273	0.259	1.077	0.833	1.392	0.583
1	rs10745353	109877506	1p13	A	0.208	0.163	1.403	0.710	2.775	0.323	A	0.261	0.263	0.987	0.491	1.981	0.984	A	0.258	0.236	1.121	1.004	1.252	0.042	A	0.273	0.259	1.077	0.833	1.392	0.583
1	rs4603158	109879549	1p13	C	0.292	0.255	1.236	0.671	2.277	0.460	C	0.272	0.329	0.743	0.370	1.493	0.396	C	0.258	0.236	1.121	1.004	1.252	0.042	C	0.273	0.259	1.077	0.833	1.392	0.583
1	rs11102972	109880721	1p13	C	0.193	0.104	2.199	0.982	4.928	0.062	C	0.217	0.204	1.327	0.628	2.805	0.458	C	0.210	0.191	1.123	0.996	1.266	0.053	C	0.216	0.216	0.998	0.759	1.314	0.985
1	rs10858084	109882250	1p13	T	0.292	0.255	1.236	0.671	2.277	0.460	T	0.272	0.329	0.743	0.370	1.493	0.396	T	0.257	0.236	1.120	1.002	1.251	0.045	T	0.273	0.259	1.077	0.833	1.392	0.583
1	rs2228604	109884775	1p13	A	0.292	0.255	1.236	0.671	2.277	0.460	A	0.272	0.329	0.743	0.370	1.493	0.396	T	0.257	0.236	1.120	1.002	1.251	0.045	T	0.273	0.259	1.077	0.833	1.392	0.583
1	rs4970843	109887191	1p13	C	0.472	0.388	1.449	0.862	2.435	0.191	C	0.457	0.454	1.196	0.644	2.221	0.579	T	0.455	0.455	0.999	0.908	1.099	0.989	C	0.495	0.500	0.981	0.781	1.232	0.855
1	rs3879448	109891423	1p13	G	0.299	0.255	1.296	0.701	2.395	0.376	G	0.294	0.342	0.758	0.385	1.492	0.417	G	0.265	0.244	1.114	0.998	1.243	0.057	G	0.287	0.271	1.086	0.842	1.402	0.523
1	rs7536292	109894693	1p13	C	0.160	0.235	0.572	0.291	1.124	0.102	C	0.185	0.151	0.954	0.410	2.219	0.912	C	0.170	0.177	0.953	0.840	1.081	0.444	C	0.179	0.202	0.861	0.648	1.144	0.304
1	rs1030522	109900707	1p13	G	0.289	0.255	1.216	0.660	2.241	0.535	G	0.272	0.329	0.743	0.370	1.493	0.396	C	0.257	0.235	1.120	1.003	1.251	0.049	C	0.273	0.259	1.077	0.833	1.392	0.583
1	rs3879449	109904971	1p13	A	0.292	0.255	1.236	0.671	2.277	0.460	A	0.272	0.329	0.743	0.370	1.493	0.396	A	0.257	0.235	1.120	1.003	1.251	0.049	A	0.273	0.259	1.077	0.833	1.392	0.583
1	rs11581665	109910013	1p13	T	0.056	0.061	0.892	0.287	2.771	0.807	T	0.054	0.105	0.479	0.141	1.632	0.219	T	0.134	0.133	1.012	0.880	1.163	0.879	T	0.158	0.131	1.251	0.908	1.725	0.202
1	rs10858086	109915075	1p13	C	0.257	0.219	1.280	0.672	2.438	0.476	C	0.261	0.296	0.811	0.405	1.625	0.502	C	0.257	0.235	1.120	1.003	1.251	0.049	C	0.273	0.259	1.077	0.833	1.392	0.583
1	rs1149175	109922377	1p13	A	0.063	0.061	1.007	0.332	3.058	0.968	A	0.065	0.112	0.558	0.175	1.778	0.329	A	0.135	0.133	1.017	0.884	1.169	0.826	A	0.158	0.131	1.251	0.908	1.725	0.202
1	rs12037569	109923677	1p13	T	0.125	0.194	0.571	0.286	1.137	0.095	T	0.196	0.118	1.583	0.654	3.831	0.308	T	0.142	0.143	0.991	0.863	1.137	0.893	T	0.158	0.169	0.924	0.684	1.248	0.616
1	rs10858089	109926828	1p13	T	0.292	0.255	1.236	0.671	2.277	0.460	T	0.272	0.329	0.743	0.370	1.493	0.396	T	0.257	0.235	1.122	1.004	1.253	0.041	T	0.273	0.259	1.077	0.833	1.392	0.583
1	rs4970752	109930852	1p13	C	0.299	0.277	1.123	0.604	2.088	0.720	C	0.283	0.336	0.817	0.409	1.631	0.560	C	0.257	0.235	1.122	1.004	1.253	0.041	C	0.273	0.259	1.077	0.833	1.392	0.583
1	rs10745354	109931908	1p13	C	0.292	0.255	1.236	0.671	2.277	0.460	C	0.272	0.329	0.743	0.370	1.493	0.396	C	0.257	0.235	1.122	1.004	1.253	0.041	C	0.273	0.259	1.077	0.833	1.392	0.583
1	rs3768494	109935427	1p13	T	0.257	0.214	1.314	0.691	2.499	0.371	T	0.261	0.296	0.811	0.405	1.625	0.502	A	0.257	0.235	1.122	1.004	1.253	0.041	A	0.273	0.259	1.077	0.833	1.392	0.583
1	rs1880670	109941133	1p13	C	0.282	0.255	1.164	0.624	2.172	0.640	C	0.272	0.329	0.743	0.370	1.493	0.396	C	0.257	0.235	1.122	1.004	1.253	0.041	C	0.273	0.259	1.077	0.833	1.392	0.583
1	rs10858092	109943893	1p13	C	0.292	0.255	1.236	0.671	2.277	0.460	C	0.272	0.329	0.743	0.370	1.493	0.396	C	0.257	0.235	1.122	1.004	1.253	0.041	C	0.273	0.259	1.077	0.833	1.392	0.583
1	rs4120621	109949008	1p13	T	0.257	0.214	1.314	0.691	2.499	0.371	T	0.261	0.296	0.811	0.405	1.625	0.502	A	0.257	0.235	1.122	1.004	1.253	0.041	A	0.273	0.259	1.077	0.833	1.392	0.583
1	rs2936033	222751034	1q41	A	0.493	0.469	1.072	0.653	1.759	0.803	A	0.359	0.408	0.786	0.414	1.490	0.489	T	0.192	0.189	1.021	0.905	1.150	0.751	T	0.186	0.183	1.020	0.767	1.356	0.889
1	rs3008647	222759007	1q41	T	0.271	0.163	1.915	0.962	3.811	0.064	T	0.163	0.171	1.221	0.537	2.780	0.628	T	0.162	0.158	1.028	0.902	1.171	0.694	T	0.165	0.156	1.066	0.788	1.442	0.651
1	rs2270705	222761771	1q41	C	0.042	0.102	0.339	0.113	1.015	0.036	C	0.152	0.053	2.794	0.929	8.410	0.070														
1	rs2936027	222762316	1q41	A	0.049	0.071	0.697	0.226	2.154	0.578	A	0.076	0.086	1.046	0.322	3.402	0.998	T	0.098	0.132	0.718	0.617	0.836	0.001	T	0.142	0.125	1.166	0.837	1.624	0.377
1	rs17464857	222762709	1q41	G	0.257	0.153	1.902	0.952	3.802	0.065	G	0.163	0.171	1.221	0.537	2.780	0.628	G	0.162	0.159	1.028	0.902	1.170	0.700	G	0.165	0.156	1.066	0.788	1.442	0.651
1	rs2936041	222762773	1q41	A	0.271	0.163	1.915	0.962	3.811	0.064	A	0.163	0.171	1.221	0.537	2.780	0.628	T	0.161	0.158	1.021	0.896	1.165	0.777	T	0.165	0.156	1.066	0.788	1.442	0.651
1	rs4846767	222763026	1q41	C	0.424	0.357	1.355	0.785	2.339	0.272	C	0.283	0.342	1.025	0.539	1.949	0.941	C	0.192	0.225	0.808	0.714	0.914	0.001	C	0.229	0.233	0.973	0.736	1.288	0.890
1	rs3008650	222763215	1q41	G	0.153	0.194	0.789	0.381	1.637	0.525	G	0.120	0.171	0.849	0.367	1.965	0.720	G	0.101	0.136	0.720	0.620	0.835	0.001	G	0.147	0.136	1.100	0.796	1.520	0.572
1	rs2936040	222763661	1q41	T	0.056	0.082	0.717	0.263	1.954	0.598	T	0.076	0.086	1.046	0.322	3.402	0.998	A	0.098	0.132	0.718	0.617	0.836	0.001	A	0.142	0.125	1.166	0.837	1.624	0.377
1	rs3008653	222764791	1q41	G	0.257	0.163	1.756	0.888	3.473	0.101	G	0.163	0.171	1.221	0.537	2.780	0.628	G	0.163	0.159	1.030	0.904	1.173	0.673	G	0.166	0.156	1.078	0.798	1.455	0.604
1	rs3008654	222766609	1q41	A	0.254	0.163	1.720	0.870	3.399	0.111	A	0.163	0.171	1.221	0.537	2.780	0.628	A	0.148	0.150	0.985	0.856	1.133	0.828	A	0.155	0.145	1.089	0.787	1.508	0.573
1	rs1995152	222769593	1q41	C	0.097	0.122	0.818	0.351	1.909	0.654	C	0.087	0.132	0.816	0.307	2.168	0.693	C	0.098	0.132	0.718	0.617	0.836	0.001	C	0.142	0.125	1.166	0.837	1.624	0.377
1	rs1909194	222770703	1q41	G	0.243	0.153	1.747	0.879	3.473	0.098	G	0.163	0.171	1.221	0.537	2.780	0.628	G	0.162	0.159	1.028	0.902	1.171	0.702	G	0.166	0.156	1.078	0.798	1.455	0.604
1	rs3002130	222772139	1q41	C	0.257	0.163	1.756	0.888	3.473	0.101	C	0.163	0.171	1.221	0.537	2.780	0.628	C	0.164	0.158	1.046	0.918	1.192	0.522	C	0.165	0.156	1.065	0.789	1.439	0.643
1	rs3002142	222788062	1q41	C	0.188	0.225	0.821	0.454	1.486	0.537	C	0.087	0.118	1.132	0.388	3.307	0.812	C	0.134	0.115	1.197	1.034	1.386	0.017	C	0.095	0.145	0.619	0.436	0.879	0.006
1	rs904323	222790366	1q41	A	0.250	0.194	1.347	0.730	2.483	0.354	A	0.152	0.197	0.802	0.347	1.849	0.611	A	0.116	0.149	0.743	0.644	0.858	0.001	A	0.144	0.137	1.059	0.766	1.465	0.732
1	rs17163303	222795118	1q41	T	0.132	0.122	1.190	0.541	2.616	0.683	T	0.109	0.105	1.249	0.474	3.288	0.658	T	0.068	0.067	1.023	0.845	1.238	0.818	T	0.066	0.082	0.792	0.516	1.215	0.300
1	rs17011666	222798965	1q41	G	0.257	0.255	0.959	0.514	1.791	0.907	G	0.185	0.283	0.690	0.302	1.577	0.385	G	0.197	0.231	0.815	0.724	0.917	0.003	G	0.223	0.219	1.022	0.786	1.330	0.882
1	rs2936052	222802376	1q41	C	0.222	0.194	1.148	0.578	2.282	0.688	C	0.174	0.250	0.845	0.363	1.964	0.696	G	0.169	0.206	0.785	0.694	0.889	0.001	G	0.190	0.192	0.989	0.748	1.309	0.951
1	rs3008621	222804046	1q41	A	0.118	0.082	1.456	0.564	3.758	0.449	A	0.120	0.138	1.052	0.389	2.847	0.875	A	0.127	0.163	0.748	0.652	0.859	0.001	A	0.155	0.155	1.006	0.742	1.364	0.965
1	rs3002145	222807013	1q41	T	0.118	0.083	1.406	0.543	3.639	0.479	T	0.120	0.138	1.052	0.389	2.847	0.875	T	0.127	0.163	0.748	0.652	0.859	0.001	T	0.155	0.155	1.006	0.742	1.364	0.965
1	rs1391557	222809616	1q41	T	0.208	0.194	1.080	0.546	2.135	0.837	T	0.207	0.237	1.303	0.567	2.994	0.530	T	0.160	0.192	0.801	0.705	0.909	0.003	T	0.173	0.189	0.898	0.676	1.194	0.454
1	rs2133188	222813753	1q41	T	0.204	0.188	1.103	0.553	2.200	0.796	T	0.200	0.237	1.225	0.522	2.872	0.656	T	0.160	0.192	0.804	0.709	0.913	0.003	T	0.174	0.189	0.908	0.683	1.207	0.522
1	rs2133189	222814442	1q41	C	0.333	0.327	1.029	0.605	1.751	0.977	C	0.294	0.355	1.045	0.533	2.048	0.909	C	0.257	0.285	0.870	0.781	0.969	0.017	C	0.269	0.298	0.877	0.690	1.114	0.314
1	rs17163358	222820639	1q41	G	0.375	0.367	1.048	0.603	1.821	0.824	G	0.315	0.375	1.035	0.523	2.045	0.943	G	0.257	0.285	0.870	0.781	0.969	0.016	G	0.268	0.298	0.870	0.685	1.106	0.285
1	rs17531063	222821709	1q41	G	0.063	0.051	1.257	0.427	3.704	0.648	G	0.076	0.059	1.229	0.386	3.915	0.812	G	0.069	0.066	1.048	0.865	1.269	0.632	G	0.065	0.082	0.771	0.501	1.186	0.251
1	rs17465637	222823529	1q41	A	0.361	0.367	0.989	0.560	1.745	0.983	A	0.315	0.375	1.035	0.523	2.045	0.943	A	0.256	0.283	0.873	0.783	0.972	0.016	A	0.269	0.297	0.884	0.695	1.123	0.344
1	rs17011681	222825088	1q41	C	0.368	0.367	1.019	0.582	1.783	0.898	C	0.315	0.375	1.035	0.523	2.045	0.943	C	0.257	0.284	0.871	0.782	0.971	0.016	C	0.269	0.298	0.877	0.690	1.114	0.314
1	rs2291832	222826481	1q41	G	0.375	0.367	1.048	0.603	1.821	0.824	G	0.315	0.375	1.035	0.523	2.045	0.943	G	0.257	0.284	0.871	0.782	0.971	0.016	G	0.269	0.298	0.877	0.690	1.114	0.314
1	rs2088514	222831372	1q41	G	0.201	0.194	1.033	0.523	2.043	0.984	G	0.207	0.237	1.303	0.567	2.994	0.530	G	0.160	0.193	0.798	0.703	0.905	0.003	G	0.173	0.189	0.898	0.676	1.194	0.454
1	rs2291834	222832295	1q41	T	0.368	0.367	1.019	0.582	1.783	0.898	T	0.315	0.375	1.035	0.523	2.045	0.943	T	0.257	0.284	0.871	0.782	0.971	0.016	T	0.269	0.298	0.877	0.690	1.114	0.314
1	rs17163384	222835222	1q41	C	0.125	0.092	1.548	0.645	3.716	0.321	C	0.098	0.099	1.040	0.378	2.860	0.813	C	0.069	0.066	1.048	0.865	1.269	0.632	C	0.065	0.082	0.771	0.501	1.186	0.251
1	rs2270707	222837594	1q41	G	0.236	0.250	0.906	0.488	1.684	0.766	G	0.217	0.270	1.044	0.465	2.344	0.913	G	0.188	0.218	0.829	0.735	0.934	0.006	G	0.207	0.218	0.941	0.722	1.225	0.667
1	rs1053316	222839838	1q41	A	0.194	0.225	0.861	0.446	1.663	0.659	A	0.120	0.171	0.713	0.308	1.647	0.404	A	0.097	0.093	1.047	0.890	1.233	0.555	A	0.097	0.110	0.864	0.599	1.247	0.475
1	rs3008633	222844840	1q41	T	0.194	0.194	1.041	0.543	1.995	0.878	T	0.109	0.151	0.744	0.301	1.839	0.494	T	0.075	0.073	1.037	0.865	1.243	0.674	T	0.079	0.096	0.808	0.545	1.197	0.304
1	rs1391558	222848029	1q41	G	0.167	0.143	1.184	0.561	2.496	0.687	G	0.196	0.204	1.521	0.642	3.604	0.348	C	0.160	0.193	0.796	0.701	0.903	0.002	C	0.173	0.189	0.898	0.676	1.194	0.454
9	rs756641	21902354	9p21	A	0.340	0.357	0.922	0.562	1.513	0.716	A	0.359	0.296	1.077	0.576	2.017	0.808														
9	rs10811634	21905379	9p21	T	0.347	0.347	0.990	0.608	1.613	0.929	T	0.348	0.280	1.083	0.575	2.041	0.814														
9	rs16938590	21909979	9p21	G	0.194	0.122	1.611	0.777	3.342	0.170	G	0.130	0.112	1.136	0.442	2.918	0.827														
9	rs4977746	21920346	9p21	C	0.375	0.327	1.240	0.701	2.192	0.465	C	0.272	0.237	1.175	0.583	2.369	0.643	C	0.166	0.170	0.971	0.856	1.102	0.669	C	0.153	0.169	0.895	0.666	1.203	0.479
9	rs10811638	21923279	9p21	G	0.125	0.061	2.134	0.795	5.728	0.122	G	0.130	0.053	1.669	0.548	5.081	0.363	G	0.166	0.171	0.967	0.852	1.097	0.629	G	0.153	0.169	0.895	0.666	1.203	0.479
9	rs7852128	21925855	9p21	G	0.347	0.296	1.260	0.706	2.249	0.449	G	0.283	0.217	1.466	0.728	2.952	0.285	G	0.172	0.174	0.987	0.871	1.118	0.863	G	0.153	0.172	0.878	0.656	1.176	0.399
9	rs10965186	21927913	9p21	A	0.111	0.061	1.857	0.688	5.007	0.221	A	0.130	0.053	1.669	0.548	5.081	0.363	A	0.172	0.174	0.987	0.871	1.118	0.863	A	0.153	0.172	0.878	0.656	1.176	0.399
9	rs2518713	21929666	9p21	G	0.097	0.061	1.562	0.575	4.245	0.396	G	0.120	0.046	1.900	0.589	6.125	0.278	G	0.175	0.177	0.989	0.873	1.120	0.882	G	0.157	0.178	0.864	0.648	1.151	0.322
9	rs7864029	21930147	9p21	C	0.333	0.296	1.169	0.659	2.075	0.612	C	0.283	0.237	1.403	0.695	2.833	0.356	C	0.179	0.182	0.977	0.864	1.105	0.727	C	0.166	0.180	0.911	0.684	1.214	0.523
9	rs7869004	21931896	9p21	T	0.333	0.296	1.169	0.659	2.075	0.612	T	0.283	0.230	1.520	0.751	3.079	0.279	T	0.178	0.181	0.983	0.868	1.112	0.796	T	0.165	0.181	0.892	0.670	1.188	0.449
9	rs4977750	21944317	9p21	C	0.347	0.306	1.147	0.668	1.971	0.611	C	0.294	0.283	0.862	0.446	1.665	0.659	C	0.137	0.131	1.048	0.908	1.209	0.525	C	0.092	0.128	0.703	0.495	0.999	0.047
9	rs2811717	21946322	9p21	C	0.438	0.388	1.187	0.690	2.041	0.513	C	0.348	0.342	0.815	0.433	1.533	0.554	C	0.137	0.132	1.047	0.908	1.208	0.527	C	0.092	0.128	0.703	0.495	0.999	0.047
9	rs2811720	21947957	9p21	G	0.299	0.225	1.369	0.765	2.450	0.281	G	0.207	0.171	0.948	0.441	2.039	0.896	G	0.140	0.138	1.021	0.888	1.175	0.762	G	0.092	0.128	0.703	0.495	0.999	0.047
9	rs10965197	21948666	9p21	T	0.345	0.375	0.860	0.507	1.459	0.552	T	0.289	0.384	0.762	0.401	1.449	0.402	T	0.396	0.395	1.008	0.914	1.112	0.884	T	0.347	0.390	0.820	0.647	1.040	0.115
9	rs10757260	21953137	9p21	A	0.389	0.418	0.921	0.534	1.588	0.783	A	0.478	0.395	1.412	0.798	2.499	0.234	G	0.413	0.397	1.068	0.969	1.177	0.178	G	0.326	0.385	0.773	0.613	0.975	0.046
9	rs10757261	21954953	9p21	G	0.389	0.418	0.921	0.534	1.588	0.783	G	0.478	0.395	1.412	0.798	2.499	0.234	A	0.413	0.397	1.068	0.969	1.177	0.178	A	0.326	0.385	0.773	0.613	0.975	0.046
9	rs12335941	21955669	9p21	A	0.382	0.418	0.896	0.521	1.542	0.719	A	0.478	0.395	1.412	0.798	2.499	0.234	G	0.414	0.398	1.067	0.968	1.175	0.184	G	0.326	0.385	0.773	0.613	0.975	0.046
9	rs717326	21958524	9p21	G	0.042	0.010	4.254	0.493	####	0.197	G	0.043	0.059	0.797	0.225	2.829	0.791	C	0.076	0.086	0.872	0.729	1.043	0.135	C	0.056	0.080	0.678	0.434	1.061	0.095
9	rs7041637	21961866	9p21	A	0.181	0.225	0.809	0.414	1.579	0.528	A	0.283	0.243	1.052	0.566	1.956	0.875	A	0.370	0.338	1.154	1.042	1.277	0.007	A	0.407	0.323	1.495	1.168	1.914	0.004
9	rs3731257	21966221	9p21	T	0.162	0.184	0.893	0.434	1.840	0.774	T	0.261	0.230	0.999	0.526	1.897	1	A	0.323	0.288	1.201	1.077	1.340	0.001	A	0.350	0.289	1.407	1.081	1.830	0.011
9	rs2518719	21970427	9p21	G	0.111	0.163	0.626	0.293	1.337	0.206	G	0.120	0.151	0.664	0.272	1.625	0.363	G	0.159	0.134	1.215	1.062	1.391	0.004	G	0.153	0.159	0.954	0.699	1.302	0.799
9	rs2811708	21973422	9p21	T	0.444	0.429	0.996	0.572	1.737	0.998	T	0.370	0.401	0.751	0.399	1.414	0.385	T	0.346	0.314	1.156	1.045	1.279	0.004	T	0.279	0.309	0.861	0.673	1.102	0.260
9	rs3731239	21974218	9p21	C	0.207	0.204	1.060	0.524	2.145	0.878	C	0.217	0.132	1.992	0.946	4.198	0.072	G	0.242	0.298	0.747	0.670	0.834	0.001	G	0.289	0.300	0.949	0.740	1.217	0.694
9	rs3731222	21983914	9p21	G	0.104	0.163	0.588	0.273	1.266	0.196	G	0.087	0.151	0.493	0.188	1.297	0.130	C	0.155	0.136	1.166	1.019	1.334	0.025	C	0.153	0.161	0.942	0.689	1.288	0.732
9	rs3731213	21986218	9p21	A	0.083	0.082	1.079	0.462	2.524	0.856	A	0.076	0.112	0.691	0.246	1.940	0.510	T	0.018	0.024	0.730	0.523	1.020	0.056	T	0.010	0.008	1.229	0.371	4.070	0.699
9	rs3731211	21986847	9p21	A	0.444	0.408	1.108	0.628	1.956	0.714	A	0.370	0.408	0.745	0.398	1.397	0.362	T	0.329	0.296	1.161	1.048	1.286	0.003	T	0.271	0.309	0.824	0.642	1.058	0.158
9	rs3731204	21987584	9p21	G	0.111	0.163	0.626	0.293	1.337	0.206	G	0.098	0.151	0.550	0.215	1.403	0.192	C	0.155	0.136	1.166	1.019	1.334	0.025	C	0.153	0.161	0.942	0.689	1.288	0.732
9	rs3731201	21988896	9p21	G	0.326	0.245	1.433	0.787	2.606	0.233	G	0.272	0.250	1.072	0.519	2.215	0.861	C	0.178	0.164	1.107	0.974	1.258	0.107	C	0.119	0.148	0.779	0.562	1.080	0.136
9	rs7036656	21990457	9p21	C	0.444	0.408	1.108	0.628	1.956	0.714	C	0.370	0.395	0.795	0.419	1.509	0.482	C	0.330	0.298	1.159	1.047	1.284	0.003	C	0.273	0.317	0.802	0.626	1.027	0.100
9	rs2811710	21991923	9p21	C	0.417	0.449	0.900	0.515	1.574	0.721	C	0.489	0.395	1.554	0.862	2.802	0.129	T	0.411	0.393	1.079	0.979	1.189	0.107	T	0.326	0.383	0.782	0.622	0.984	0.045

9	rs2811711	21993964	9p21	C	0.194	0.194	0.964	0.495	1.876	0.915	C	0.141	0.184	0.715	0.308	1.661	0.489	C	0.155	0.138	1.144	1.000	1.310	0.049	C	0.158	0.172	0.900	0.662	1.225	0.526
9	rs3218020	21997872	9p21	T	0.389	0.388	0.995	0.590	1.679	0.968	T	0.413	0.434	0.804	0.442	1.462	0.502	A	0.467	0.420	1.211	1.099	1.333	0.001	A	0.484	0.409	1.374	1.091	1.729	0.005
9	rs3218002	22000841	9p21	T	0.250	0.184	1.471	0.784	2.763	0.233	T	0.207	0.178	1.005	0.463	2.181	0.999	A	0.131	0.119	1.113	0.963	1.288	0.151	A	0.082	0.106	0.770	0.531	1.117	0.200
9	rs3217992	22003223	9p21	A	0.410	0.418	0.972	0.587	1.609	0.973	A	0.435	0.447	0.850	0.474	1.525	0.587	T	0.502	0.458	1.192	1.084	1.312	0.001	T	0.507	0.439	1.339	1.063	1.688	0.009
9	rs3217986	22005330	9p21	C	0.042	0.000	#####	0	inf	0.959	C	0.043	0.059	0.797	0.225	2.829	0.750	G	0.068	0.079	0.847	0.703	1.022	0.091	G	0.052	0.065	0.779	0.483	1.258	0.312
9	rs974336	22006348	9p21	A	0.243	0.184	1.402	0.749	2.625	0.360	A	0.207	0.178	1.005	0.463	2.181	0.999	T	0.131	0.118	1.120	0.968	1.296	0.131	T	0.082	0.106	0.770	0.531	1.117	0.200
9	rs2069422	22008026	9p21	C	0.222	0.135	1.861	0.910	3.807	0.079	C	0.174	0.138	1.019	0.442	2.349	0.956	G	0.133	0.122	1.107	0.957	1.281	0.181	G	0.084	0.107	0.766	0.526	1.115	0.173
9	rs2069418	22009698	9p21	C	0.201	0.296	0.586	0.319	1.076	0.082	C	0.294	0.211	1.864	0.942	3.688	0.072	G	0.320	0.364	0.824	0.745	0.911	0.001	G	0.376	0.412	0.866	0.692	1.085	0.221
9	rs575427	22011477	9p21	C	0.076	0.115	0.618	0.242	1.578	0.288	C	0.033	0.072	0.423	0.095	1.881	0.229	G	0.083	0.078	1.075	0.902	1.280	0.408	G	0.100	0.112	0.883	0.613	1.271	0.509
9	rs10811640	22013411	9p21	G	0.438	0.429	1.035	0.620	1.728	0.912	G	0.511	0.368	1.760	0.962	3.219	0.068	G	0.451	0.483	0.879	0.799	0.967	0.013	G	0.457	0.517	0.781	0.623	0.979	0.035
9	rs643319	22017836	9p21	T	0.382	0.316	1.275	0.767	2.118	0.349	T	0.413	0.290	1.496	0.812	2.755	0.199	A	0.370	0.390	0.917	0.832	1.012	0.090	A	0.387	0.434	0.819	0.649	1.032	0.088
9	rs7044859	22018781	9p21	T	0.438	0.429	1.035	0.620	1.728	0.912	T	0.511	0.362	1.773	0.971	3.238	0.070	T	0.451	0.484	0.879	0.800	0.967	0.012	T	0.457	0.517	0.781	0.623	0.979	0.035
9	rs523096	22019129	9p21	C	0.188	0.245	0.710	0.382	1.319	0.287	C	0.304	0.191	2.072	1.030	4.165	0.042	G	0.317	0.361	0.821	0.742	0.909	0.001	G	0.373	0.402	0.888	0.708	1.113	0.313
9	rs518394	22019673	9p21	G	0.188	0.245	0.710	0.382	1.319	0.287	G	0.294	0.191	1.981	0.987	3.977	0.065	C	0.315	0.359	0.822	0.743	0.910	0.001	C	0.369	0.404	0.870	0.694	1.091	0.228
9	rs10757264	22019732	9p21	A	0.409	0.388	1.084	0.648	1.815	0.800	A	0.424	0.342	1.360	0.736	2.515	0.321	A	0.408	0.433	0.902	0.819	0.993	0.044	A	0.410	0.470	0.782	0.623	0.981	0.031
9	rs10965212	22023795	9p21	T	0.417	0.388	1.103	0.667	1.825	0.695	T	0.446	0.347	1.423	0.782	2.592	0.250	T	0.417	0.447	0.888	0.807	0.978	0.022	T	0.431	0.484	0.797	0.633	1.004	0.054
9	rs496892	22024351	9p21	A	0.382	0.323	1.239	0.744	2.064	0.391	A	0.370	0.287	1.261	0.673	2.361	0.475	C	0.368	0.389	0.913	0.827	1.007	0.070	C	0.382	0.432	0.805	0.637	1.017	0.065
9	rs10738604	22025493	9p21	A	0.319	0.367	0.816	0.468	1.421	0.490	A	0.413	0.408	0.879	0.483	1.600	0.700	A	0.484	0.439	1.195	1.086	1.315	0.002	A	0.495	0.421	1.366	1.085	1.719	0.005
9	rs1591136	22026834	9p21	C	0.417	0.388	1.103	0.667	1.825	0.695	C	0.446	0.349	1.420	0.779	2.588	0.252	G	0.417	0.446	0.889	0.808	0.978	0.026	G	0.431	0.484	0.797	0.633	1.004	0.054
9	rs598664	22027551	9p21	G	0.203	0.133	1.676	0.821	3.422	0.142	G	0.174	0.138	1.019	0.442	2.349	0.956	C	0.129	0.120	1.094	0.945	1.267	0.219	C	0.084	0.109	0.753	0.517	1.096	0.149
9	rs7049105	22028801	9p21	A	0.417	0.388	1.103	0.667	1.825	0.695	A	0.446	0.349	1.420	0.779	2.588	0.252	A	0.415	0.447	0.881	0.801	0.969	0.016	A	0.431	0.484	0.797	0.633	1.004	0.054
9	rs10965215	22029445	9p21	A	0.493	0.448	1.188	0.710	1.986	0.514	G	0.446	0.434	1.069	0.603	1.897	0.813	G	0.422	0.455	0.876	0.797	0.964	0.010	G	0.437	0.491	0.798	0.634	1.005	0.052
9	rs564398	22029547	9p21	G	0.167	0.204	0.767	0.401	1.466	0.432	G	0.239	0.171	1.703	0.803	3.610	0.162	C	0.282	0.324	0.823	0.742	0.914	0.002	C	0.347	0.372	0.897	0.711	1.132	0.367
9	rs662463	22030438	9p21	T	0.211	0.133	1.782	0.872	3.640	0.105	T	0.185	0.145	1.067	0.466	2.442	0.862	A	0.124	0.113	1.111	0.957	1.289	0.163	A	0.079	0.106	0.733	0.500	1.073	0.129
9	rs7865618	22031005	9p21	G	0.167	0.214	0.716	0.375	1.367	0.307	G	0.239	0.171	1.703	0.803	3.610	0.162	G	0.289	0.331	0.823	0.742	0.912	0.001	G	0.352	0.380	0.886	0.703	1.117	0.312
9	rs10115049	22032119	9p21	A	0.479	0.490	0.962	0.577	1.603	0.908	A	0.457	0.434	1.106	0.621	1.972	0.729	A	0.417	0.448	0.882	0.802	0.971	0.021	A	0.432	0.484	0.802	0.637	1.010	0.065
9	rs2157719	22033366	9p21	G	0.167	0.204	0.767	0.401	1.466	0.432	G	0.233	0.171	1.652	0.779	3.503	0.225	C	0.288	0.330	0.821	0.741	0.911	0.002	C	0.350	0.382	0.875	0.694	1.102	0.263
9	rs1008878	22036112	9p21	G	0.167	0.225	0.692	0.369	1.298	0.240	G	0.239	0.171	1.703	0.803	3.610	0.162	G	0.288	0.330	0.821	0.741	0.911	0.002	G	0.350	0.382	0.875	0.694	1.102	0.263
9	rs12376000	22039426	9p21	T	0.076	0.133	0.532	0.224	1.267	0.175	T	0.054	0.072	0.728	0.226	2.343	0.526	T	0.084	0.079	1.061	0.892	1.262	0.502	T	0.100	0.110	0.895	0.620	1.294	0.567
9	rs17694493	22041998	9p21	G	0.215	0.125	1.962	0.944	4.076	0.068	G	0.185	0.138	1.194	0.517	2.758	0.691	G	0.147	0.138	1.080	0.941	1.241	0.254	G	0.118	0.136	0.853	0.612	1.189	0.357
9	rs12352425	22042086	9p21	A	0.069	0.041	1.821	0.533	6.220	0.340	A	0.065	0.112	0.707	0.246	2.031	0.528	A	0.070	0.081	0.855	0.711	1.028	0.101	A	0.052	0.069	0.726	0.455	1.159	0.187
9	rs1412829	22043926	9p21	C	0.167	0.214	0.716	0.375	1.367	0.307	C	0.239	0.171	1.703	0.803	3.610	0.162	G	0.286	0.327	0.827	0.745	0.917	0.003	G	0.352	0.383	0.875	0.694	1.102	0.250
9	rs1360589	22045317	9p21	G	0.160	0.174	0.870	0.448	1.689	0.686	G	0.228	0.165	1.648	0.776	3.498	0.205	C	0.288	0.329	0.828	0.746	0.917	0.003	C	0.347	0.383	0.858	0.681	1.079	0.190
9	rs7028570	22048683	9p21	A	0.500	0.500	1.007	0.610	1.665	0.928	G	0.435	0.421	1.057	0.594	1.879	0.915	G	0.418	0.448	0.887	0.806	0.976	0.017	G	0.429	0.483	0.799	0.636	1.005	0.055
9	rs17756311	22053895	9p21	A	0.215	0.122	1.975	0.953	4.092	0.066	A	0.174	0.138	1.019	0.442	2.349	0.956	A	0.110	0.100	1.113	0.952	1.301	0.194	A	0.074	0.088	0.831	0.557	1.240	0.395
9	rs17694572	22054356	9p21	A	0.215	0.122	1.975	0.953	4.092	0.066	A	0.174	0.138	1.019	0.442	2.349	0.956	A	0.111	0.098	1.140	0.974	1.333	0.103	A	0.077	0.090	0.852	0.574	1.265	0.464
9	rs10120688	22056499	9p21	A	0.500	0.480	1.102	0.655	1.855	0.711	G	0.457	0.428	1.066	0.609	1.868	0.803	G	0.410	0.442	0.881	0.801	0.970	0.016	G	0.431	0.464	0.871	0.693	1.095	0.243
9	rs1537378	22061614	9p21	T	0.155	0.174	0.846	0.435	1.646	0.629	T	0.228	0.165	1.648	0.776	3.498	0.205	A	0.268	0.312	0.811	0.730	0.900	0.001	A	0.319	0.366	0.821	0.652	1.034	0.098
9	rs1011970	22062134	9p21	T	0.299	0.174	2.155	1.106	4.198	0.021	T	0.261	0.243	0.923	0.485	1.755	0.822	T	0.184	0.181	1.023	0.903	1.159	0.710	T	0.129	0.159	0.786	0.576	1.073	0.128
9	rs8181047	22064465	9p21	A	0.090	0.071	1.218	0.466	3.180	0.701	A	0.185	0.099	2.574	1.020	6.499	0.041	A	0.192	0.239	0.764	0.680	0.858	0.001	A	0.236	0.265	0.860	0.668	1.107	0.259
9	rs10811647	22065002	9p21	G	0.431	0.459	0.909	0.537	1.539	0.743	G	0.457	0.461	0.975	0.555	1.710	0.902	C	0.464	0.508	0.839	0.762	0.923	0.001	C	0.452	0.532	0.714	0.567	0.898	0.004
9	rs10965224	22067276	9p21	T	0.236	0.327	0.636	0.359	1.127	0.124	T	0.239	0.243	1.137	0.560	2.308	0.720	T	0.270	0.316	0.806	0.726	0.894	0.001	T	0.321	0.366	0.827	0.657	1.041	0.106
9	rs16905599	22069144	9p21	A	0.090	0.041	2.490	0.756	8.203	0.163	A	0.087	0.105	0.909	0.327	2.525	0.822	A	0.064	0.075	0.834	0.689	1.010	0.066	A	0.042	0.073	0.559	0.342	0.916	0.016
9	rs9632884	22072301	9p21	G	0.188	0.174	1.052	0.554	2.001	0.885	G	0.272	0.230	1.325	0.672	2.613	0.384	G	0.350	0.406	0.791	0.717	0.873	0.001	G	0.368	0.434	0.768	0.614	0.960	0.018
9	rs10757270	22072719	9p21	G	0.465	0.510	0.865	0.517	1.447	0.585	A	0.522	0.520	0.910	0.511	1.619	0.729	A	0.463	0.506	0.842	0.765	0.927	0.002	A	0.452	0.522	0.749	0.598	0.939	0.012
9	rs16923583	22073334	9p21	A	0.085	0.143	0.547	0.243	1.233	0.132	A	0.033	0.072	0.604	0.160	2.276	0.460	A	0.022	0.023	0.946	0.685	1.306	0.718	A	0.008	0.014	0.565	0.187	1.705	0.364
9	rs1412832	22077543	9p21	C	0.104	0.082	1.277	0.513	3.180	0.624	C	0.185	0.105	2.096	0.874	5.024	0.086	C	0.203	0.242	0.803	0.717	0.900	0.001	C	0.236	0.267	0.855	0.666	1.099	0.235
9	rs6475606	22081850	9p21	C	0.194	0.194	0.969	0.522	1.797	0.920	C	0.250	0.257	1.076	0.552	2.096	0.807	C	0.370	0.414	0.836	0.758	0.922	0.001	C	0.374	0.437	0.776	0.619	0.971	0.028
9	rs1547704	22082340	9p21	A	0.104	0.102	1.000	0.423	2.363	0.997	A	0.054	0.086	0.540	0.156	1.872	0.363	A	0.015	0.021	0.738	0.513	1.062	0.105	A	0.010	0.016	0.608	0.218	1.694	0.396
9	rs10965228	22082380	9p21	G	0.063	0.102	0.576	0.225	1.473	0.264	G	0.022	0.092	0.350	0.078	1.577	0.157	G	0.091	0.087	1.056	0.893	1.249	0.555	G	0.090	0.106	0.850	0.590	1.223	0.376
9	rs1333040	22083404	9p21	C	0.208	0.204	1.000	0.517	1.935	0.999	C	0.250	0.257	1.074	0.556	2.075	0.806	C	0.286	0.338	0.792	0.714	0.877	0.001	C	0.290	0.334	0.811	0.637	1.034	0.095
9	rs7857345	22087473	9p21	T	0.097	0.092	1.009	0.430	2.368	0.980	T	0.163	0.099	1.608	0.669	3.864	0.313	T	0.188	0.227	0.796	0.708	0.894	0.002	T	0.224	0.256	0.843	0.650	1.094	0.220
9	rs10757272	22088260	9p21	C	0.417	0.429	0.919	0.541	1.561	0.765	C	0.402	0.434	0.878	0.465	1.657	0.681	C	0.383	0.437	0.805	0.731	0.887	0.001	C	0.395	0.457	0.775	0.618	0.971	0.028
9	rs4977574	22098574	9p21	A	0.451	0.500	0.797	0.477	1.331	0.409	A	0.391	0.474	0.757	0.409	1.402	0.398	A	0.382	0.435	0.808	0.733	0.889	0.001	A	0.392	0.461	0.751	0.598	0.943	0.017
9	rs2891168	22098619	9p21	A	0.471	0.521	0.762	0.444	1.307	0.349	A	0.391	0.474	0.757	0.409	1.402	0.398	A	0.383	0.435	0.809	0.735	0.891	0.001	A	0.392	0.461	0.751	0.598	0.943	0.018
9	rs1537371	22099568	9p21	C	0.188	0.194	0.931	0.495	1.753	0.826	C	0.239	0.243	0.941	0.479	1.849	0.882	C	0.372	0.423	0.816	0.741	0.898	0.001	C	0.387	0.456	0.754	0.602	0.946	0.019
9	rs7859727	22102165	9p21	C	0.271	0.316	0.779	0.440	1.381	0.399	C	0.283	0.316	0.871	0.459	1.652	0.676	C	0.382	0.434	0.808	0.734	0.890	0.001	C	0.390	0.462	0.743	0.592	0.932	0.016
9	rs1333042	22103813	9p21	A	0.188	0.194	0.931	0.495	1.753	0.826	A	0.239	0.237	0.954	0.486	1.875	0.885	A	0.346	0.398	0.804	0.728	0.887	0.001	A	0.358	0.437	0.725	0.579	0.909	0.009
9	rs7859362	22105927	9p21	T	0.188	0.194	0.931	0.495	1.753	0.826	T	0.239	0.237	0.954	0.486	1.875	0.885	T	0.372	0.423	0.816	0.741	0.898	0.001	T	0.387	0.454	0.759	0.606	0.952	0.020
9	rs1333043	22106731	9p21	T	0.188	0.194	0.931	0.495	1.753	0.826	T	0.239	0.237	0.954	0.486	1.875	0.885	T	0.372	0.423	0.816	0.741	0.898	0.001	T	0.387	0.456	0.754	0.602	0.946	0.018
9	rs1412834	22110131	9p21	T	0.188	0.194	0.931	0.495	1.753	0.826	T	0.239	0.237	0.954	0.486	1.875	0.885	T	0.372	0.423	0.816	0.741	0.898	0.001	T	0.387	0.456	0.754	0.602	0.946	0.018
9	rs7341786	22112241	9p21	A	0.188	0.194	0.931	0.495	1.753	0.826	A	0.239	0.237	0.954	0.486	1.875	0.885	A	0.372	0.423	0.816	0.742	0.899	0.001	A	0.387	0.454	0.758	0.605	0.951	0.020
9	rs10733376	22114469	9p21	G	0.188	0.194	0.931	0.495	1.753	0.826	G	0.239	0.237	0.954	0.486	1.875	0.885	G	0.371	0.418	0.832	0.756	0.916	0.001	G	0.379	0.454	0.730	0.580	0.917	0.010
9	rs2383206	22115026	9p21	A	0.347	0.357	0.922	0.530	1.602	0.783	A	0.348	0.375	0.890	0.467	1.697	0.770	A	0.372	0.418	0.833	0.757	0.917	0.001	A	0.379	0.454	0.730	0.580	0.917	0.010
9	rs2383207	22115959	9p21	A	0.190	0.194	0.946	0.502	1.783	0.865	A	0.239	0.237	0.954	0.486	1.875	0.885	A	0.372	0.418	0.833	0.757	0.917	0.001	A	0.379	0.453	0.733	0.583	0.923	0.011
9	rs1333045	22119195	9p21	T	0.417	0.347	1.306	0.774	2.201	0.347	T	0.402	0.428	0.883	0.460	1.696	0.804	T	0.396	0.438	0.845	0.768	0.931	0.001	T	0.402	0.483	0.713	0.567	0.896	0.007
9	rs10738610	22123766	9p21	A	0.451	0.490	0.828	0.498	1.377	0.485	A	0.391	0.467	0.768	0.416	1.418	0.432	A	0.378	0.427	0.820	0.745	0.904	0.001	A	0.382	0.456	0.732	0.581	0.921	0.010
9	rs10757278	22124477	9p21	G	0.493	0.459	1.173	0.699	1.968	0.566	A	0.424	0.540	0.676	0.361	1.267	0.252	A	0.418	0.458	0.852	0.774	0.937	0.003	A	0.413	0.491	0.722	0.574	0.908	0.008
9	rs1333048	22125347	9p21	A	0.424	0.418	0.980	0.576	1.667	0.928	A	0.380	0.421	0.827	0.434	1.574	0.590	A	0.379	0.428	0.817	0.741	0.900	0.001	A	0.382	0.462	0.713	0.566	0.897	0.006
9	rs1333049	22125503	9p21	C	0.493	0.449	1.220	0.723	2.059	0.454	G	0.424	0.533	0.682	0.363	1.282	0.251	G	0.417	0.459	0.846	0.768	0.931	0.001	G	0.415	0.491	0.726	0.577	0.914	0.008
9	rs10757281	22127613	9p21	T	0.243	0.265	0.770	0.392	1.510	0.472	T	0.109	0.211	0.386	0.155	0.964	0.046	T	0.093	0.083	1.154	0.968	1.376	0.118	T	0.134	0.128	1.062	0.752	1.500	0.744
9	rs12347779	22128709	9p21	G	0.152	0.188	0.767	0.370	1.586	0.529	G	0.152	0.099	1.623	0.632	4.172	0.307	G	0.057	0.052	1.087	0.880	1.343	0.461	G	0.074	0.082	0.900	0.601	1.348	0.612
9	rs10965243	22130065	9p21	G	0.076	0.112	0.617	0.253	1.506	0.272	G	0.043	0.118	0.198	0.055	0.711	0.011	G	0.082	0.077	1.073	0.898	1.282	0.458	G	0.107	0.109	0.975	0.680	1.399	0.926
9	rs10965245	22130515	9p21	A	0.070	0.112	0.552	0.221	1.378	0.190	A	0.033	0.118	0.150	0.037	0.609	0.004	A	0.082	0.077	1.073	0.898	1.282	0.458	A	0.107	0.109	0.975	0.680	1.399	0.926
9	rs2891169	22131825	9p21	G	0.437	0.531	0.589	0.336	1.034	0.070	G	0.435	0.447	0.815	0.423	1.571	0.556	G	0.433	0.423	1.040	0.943	1.147	0.434	G	0.439	0.456	0.937	0.753	1.166	0.561
9	rs2383208	22132076	9p21	G	0.160	0.194	0.751	0.359	1.567	0.367	G	0.141	0.204	0.520	0.234	1.155	0.087	G	0.204	0.200	1.028	0.914	1.157	0.644	G	0.207	0.196	1.074	0.811	1.423	0.638
9	rs7045889	22133251	9p21	G	0.375	0.316	1.350	0.779	2.341	0.264	G	0.380	0.428	0.879	0.483	1.599	0.673	G	0.331	0.338	0.967	0.872	1.072	0.525	G	0.357	0.355	1.006	0.803	1.261	0.973
9	rs10811659	22133716	9p21	C	0.201	0.184	1.224	0.620	2.417	0.574	C	0.207	0.217	1.232	0.567	2.678	0.595	C	0.241	0.247	0.967	0.864	1.081	0.557	C	0.232	0.216	1.104	0.840	1.452	0.480
9	rs10757282	22133984	9p21	C	0.451	0.520	0.650	0.366	1.153	0.141	C	0.435	0.395	0.965	0.532	1.751	0.888	C	0.474	0.469	1.019	0.925	1.123	0.703	C	0.474	0.483	0.968	0.775	1.209	0.787
9	rs10811661	22134094	9p21	C	0.153	0.174	0.806	0.380	1.711	0.561	C	0.130	0.171	0.617	0.277	1.375	0.229	C	0.199	0.195	1.024	0.908	1.155	0.710	C	0.198	0.188	1.074	0.807	1.429	0.634
9	rs10757283	22134172	9p21	T	0.409	0.510	0.608	0.350	1.056	0.075	T	0.457	0.415	1.002	0.544	1.847	0.993	T	0.475	0.471	1.018	0.924	1.122	0.715	T	0.469	0.480	0.962	0.770	1.201	0.731
9	rs1333051	22136489	9p21	T	0.125	0.184	0.557	0.257	1.208	0.131	T	0.076	0.138	0.344	0.123	0.968	0.035	T	0.156	0.156	1.001	0.877	1.144	0.987	T	0.166	0.148	1.153	0.843	1.577	0.376
9	rs7018475	22137685	9p21	G	0.319	0.378	0.721	0.416	1.249	0.258	G	0.367	0.276	1.397	0.708	2.756	0.342	G	0.302	0.298	1.019	0.918	1.132	0.711	G	0.286	0.300	0.937	0.733	1.196	0.609
9	rs11791416	22138105	9p21	G	0.229	0.204	1.279	0.663	2.467	0.498	G	0.217	0.250	1.096	0.512	2.346	0.821	G	0.290	0.302	0.944	0.850	1.048	0.278	G	0.289	0.260	1.160	0.899	1.498	0.288
9	rs4977761	22138762	9p21	T	0.410	0.337	1.355	0.806	2.278	0.232	T	0.380	0.408	0.823	0.449	1.510	0.573	T	0.285	0.282	1.017	0.914	1.131	0.763	T	0.298	0.311	0.943	0.743	1.195	0.650
9	rs2065501	22140224	9p21	A	0.250	0.265	0.947	0.531	1.689	0.827	A	0.304	0.329	0.851	0.422	1.713	0.649														
9	rs4977577	22141875	9p21	C	0.444	0.469	0.877	0.516	1.490	0.623	C	0.478	0.388	1.352	0.742	2.464	0.317	C	0.275	0.278	0.984	0.877	1.104	0.781	C	0.310	0.322	0.936	0.719	1.219	0.618
9	rs7849199	22143293	9p21	A	0.181	0.188	1.003	0.530	1.899	0.990	A	0.152	0.243	0.583	0.269	1.263	0.158	A	0.292	0.303	0.952	0.857	1.057	0.373	A	0.297	0.270	1.141	0.893	1.457	0.302
9	rs2065500	22145694	9p21	G	0.174	0.102	1.925	0.843	4.395	0.099	G	0.141	0.197	0.640	0.282	1.453	0.308	G	0.216	0.219	0.984	0.877	1.104	0.782	G	0.213	0.235	0.877	0.672	1.145	0.325
9	rs7022662	22147715	9p21	G	0.174	0.102	1.925	0.843	4.395	0.099	G	0.141	0.197	0.640	0.282	1.453	0.308	G	0.221	0.223	0.992	0.884	1.113	0.896	G	0.215	0.238	0.870	0.667	1.135	0.308
9	rs12341394	22148055	9p21	T	0.194	0.214	0.946	0.512	1.747	0.839	T	0.163	0.250	0.617	0.289	1.319	0.212	T	0.293	0.304	0.950	0.855	1.054	0.347	T	0.298	0.271	1.141	0.893	1.457	0.302
9	rs7856219	22150261	9p21	C	0.150	0.167	0.971	0.492	1.919	0.934	C	0.163	0.237	0.713	0.322	1.581	0.369	C	0.297	0.309	0.946	0.852	1.050	0.305	C	0.302	0.278	1.123	0.880	1.434	0.333
9	rs10965256	22151465	9p21	A	0.028	0.071	0.348	0.095	1.272	0.062	A	0.109	0.086	1.381	0.513	3.719	0.518	A	0.075	0.072	1.042	0.868	1.252	0.684	A	0.065	0.074	0.863	0.557	1.337	0.544
9	rs7853123	22153360	9p21	G	0.458	0.438	1.140	0.665	1.955	0.617	A	0.554	0.441	1.675	0.906	3.097	0.098	A	0.420	0.413	1.032	0.936	1.138	0.520	A	0.424	0.424	1.002	0.797	1.260	0.990
9	rs944802	22155709	9p21	A	0.111	0.051	2.344	0.825	6.657	0.079	A	0.109	0.158	0.640	0.254	1.614	0.344	T	0.201	0.204	0.983	0.874	1.105	0.773	T	0.210	0.224	0.924	0.715	1.193	0.540
9	rs7028213	22157360	9p21	G	0.201	0.214	0.979	0.523	1.833	0.950	G	0.174	0.270	0.579	0.272	1.235	0.146	G	0.294	0.305	0.949	0.855	1.054	0.337	G	0.298	0.271	1.140	0.893	1.457	0.299
9	rs12238587	22158168	9p21	A	0.174	0.112	1.744	0.808	3.765	0.148	A	0.141	0.184	0.693	0.290	1.656	0.448	A	0.219	0.221	0.989	0.882	1.109	0.843	A	0.219	0.243	0.883	0.686	1.136	0.326
9	rs10757288	22159416	9p21	T	0.438	0.418	1.128	0.669	1.900	0.638	C	0.587	0.434	1.910	1.021	3.574	0.037	C	0.414	0.405	1.040	0.943	1.148	0.412	C	0.431	0.429	1.010	0.803	1.271	0.922
9	rs7864275	22161212	9p21	T	0.236	0.271	0.874	0.475	1.608	0.653	T	0.261	0.362	0.635	0.321	1.254	0.191	T	0.371	0.378	0.973	0.881	1.075	0.611	T	0.350	0.330	1.093	0.866	1.381	0.447
9	rs10965266	22161494	9p21	G	0.215	0.143	1.789	0.865	3.701	0.091	G	0.174	0.211	0.746	0.328	1.694	0.491	G	0.218	0.218	1.001	0.892	1.123	0.977	G	0.218	0.235	0.911	0.707	1.173	0.476
9	rs10965267	22161828	9p21	G	0.035	0.061	0.531	0.151	1.861	0.239	G	0.109	0.099	1.137	0.433	2.982	0.689	G	0.085	0.081	1.057	0.888	1.257	0.540	G	0.058	0.060	0.966	0.607	1.537	0.860
9	rs10811668	22164991	9p21	A	0.243	0.204	1.260	0.670	2.369	0.425	A	0.250	0.296	0.732	0.370	1.448	0.335	A	0.279	0.270	1.046	0.939	1.165	0.406	A	0.247	0.252	0.971	0.758	1.244	0.811
9	rs2779748	22166769	9p21	G	0.444	0.459	0.976	0.586	1.624	0.925	A	0.565	0.447	1.580	0.852	2.930	0.149	T	0.421	0.416	1.022	0.926	1.127	0.693	T	0.447	0.448	1.000	0.796	1.255	0.998
9	rs7863846	22168128	9p21	T	0.194	0.204	0.995	0.522	1.897	0.983	T	0.152	0.263	0.560	0.260	1.207	0.136	T	0.286	0.298	0.942	0.848	1.047	0.294	T	0.284	0.268	1.081	0.843	1.388	0.549
9	rs828580	22168464	9p21	T	0.104	0.102	1.092	0.459	2.597	0.844	T	0.109	0.105	1.088	0.448	2.643	0.860	T	0.077	0.072	1.078	0.899	1.292	0.433	T	0.048	0.054	0.899	0.549	1.472	0.662
9	rs1537377	22169700	9p21	C	0.250	0.296	0.826	0.440	1.553	0.553	C	0.228	0.349	0.653	0.336	1.271	0.211	C	0.363	0.368	0.976	0.883	1.079	0.646	C	0.332	0.320	1.055	0.832	1.337	0.660
9	rs954399	22170983	9p21	T	0.194	0.204	0.995	0.522	1.897	0.983	T	0.144	0.263	0.536	0.246	1.164	0.107	A	0.286	0.297	0.951	0.856	1.056	0.365	A	0.279	0.263	1.081	0.842	1.389	0.552
9	rs828582	22172259	9p21	A	0.347	0.357	1.009	0.578	1.763	0.978	A	0.304	0.420	0.554	0.284	1.080	0.083	A	0.380	0.382	0.992	0.899	1.095	0.877	A	0.339	0.325	1.062	0.839	1.346	0.604
9	rs10965278	22174712	9p21	A	0.194	0.174	1.090	0.554	2.145	0.817	A	0.174	0.191	0.727	0.352	1.499	0.405	A	0.272	0.269	1.017	0.913	1.132	0.748	A	0.271	0.263	1.038	0.811	1.329	0.758
9	rs10965279	22175188	9p21	C	0.056	0.061	0.892	0.287	2.771	0.784	C	0.043	0.105	0.385	0.104	1.420	0.130	C	0.068	0.070	0.971	0.803	1.174	0.724	C	0.069	0.066	1.049	0.672	1.637	0.849
9	rs10757292	22176961	9p21	T	0.111	0.082	1.482	0.576	3.814	0.362	T	0.120	0.112	0.938	0.396	2.226	0.917														
9	rs866666	22183781	9p21	T	0.507	0.429	1.368	0.800	2.338	0.239	C	0.511	0.434	1.404	0.793	2.487	0.197	C	0.321	0.334	0.935	0.841	1.040	0.208	C	0.366	0.377	0.950	0.741	1.218	0.687
9	rs2767409	22184997	9p21	A	0.265	0.239	1.179	0.640	2.172	0.587	A	0.311	0.342	1.023	0.543	1.931	0.926	A	0.352	0.349	1.016	0.915	1.130	0.756	A	0.300	0.304	0.975	0.754	1.260	0.840
9	rs1095904	22187074	9p21	T	0.222	0.174	1.364	0.724	2.569	0.329	T	0.272	0.237	1.155	0.593	2.249	0.667														
9	rs828576	22191189	9p21	C	0.529	0.438	1.482	0.848	2.590	0.153	T	0.500	0.399	1.590	0.882	2.867	0.119	T	0.320	0.331	0.944	0.848	1.051	0.283	T	0.358	0.371	0.940	0.730	1.210	0.607
9	rs2219849	22195820	9p21	C	0.493	0.490	1.010	0.616	1.655	0.993	T	0.500	0.493	1.103	0.617	1.970	0.754														
9	rs1751449	22196863	9p21	G	0.292	0.265	1.149	0.640	2.061	0.540	G	0.326	0.395	0.769	0.416	1.422	0.385	G	0.374	0.371	1.012	0.910	1.125	0.810	G	0.324	0.338	0.929	0.720	1.199	0.546
9	rs12375458	22198149	9p21	A	0.104	0.061	1.749	0.655	4.671	0.350	A	0.163	0.072	1.954	0.769	4.965	0.164														
10	rs1482472	44682973	10q11	C	0.444	0.365	1.399	0.805	2.432	0.252	C	0.367	0.415	0.703	0.378	1.308	0.278	C	0.375	0.395	0.915	0.827	1.013	0.076	C	0.389	0.377	1.052	0.838	1.320	0.648
10	rs12415866	44686664	10q11	G	0.201	0.174	1.216	0.616	2.398	0.507	G	0.141	0.197	0.419	0.177	0.990	0.032	G	0.124	0.143	0.841	0.729	0.970	0.015	G	0.107	0.107	0.996	0.686	1.445	0.989
10	rs7917089	44688587	10q11	A	0.201	0.174	1.216	0.616	2.398	0.507	A	0.141	0.197	0.419	0.177	0.991	0.032	A	0.121	0.145	0.815	0.707	0.940	0.005	A	0.098	0.103	0.957	0.654	1.400	0.829
10	rs1623851	44691241	10q11	G	0.229	0.194	1.252	0.651	2.407	0.452	G	0.185	0.250	0.496	0.239	1.030	0.056	G	0.177	0.197	0.876	0.774	0.991	0.036	G	0.158	0.170	0.911	0.669	1.239	0.560
10	rs1627329	44691633	10q11	C	0.229	0.194	1.252	0.651	2.407	0.452	C	0.185	0.250	0.496	0.239	1.030	0.056	C	0.177	0.197	0.878	0.776	0.993	0.040	C	0.158	0.170	0.911	0.669	1.239	0.560
10	rs10508883	44693544	10q11	C	0.056	0.041	1.276	0.393	4.143	0.755	C	0.043	0.072	0.380	0.099	1.460	0.133	C	0.026	0.021	1.260	0.918	1.729	0.152	C	0.018	0.021	0.857	0.378	1.944	0.768
10	rs7900182	44693742	10q11	C	0.149	0.094	1.655	0.716	3.823	0.244	G	0.144	0.100	2.103	0.746	5.931	0.193	G	0.175	0.168	1.046	0.922	1.187	0.468	G	0.207	0.181	1.162	0.885	1.525	0.305
10	rs11597731	44694201	10q11	C	0.215	0.153	1.530	0.779	3.006	0.206	T	0.207	0.138	1.942	0.833	4.530	0.099	T	0.178	0.178	1.003	0.885	1.137	0.971	T	0.210	0.191	1.119	0.854	1.464	0.447
10	rs7902040	44695308	10q11	G	0.083	0.102	0.760	0.324	1.782	0.521	G	0.043	0.079	0.255	0.068	0.951	0.024	G	0.102	0.121	0.820	0.704	0.954	0.008	G	0.089	0.079	1.148	0.764	1.724	0.497
10	rs11238911	44695862	10q11	A	0.063	0.092	0.637	0.245	1.655	0.358	A	0.033	0.079	0.188	0.045	0.789	0.007	A	0.100	0.120	0.816	0.700	0.951	0.008	A	0.089	0.077	1.174	0.780	1.766	0.431
10	rs11238913	44695973	10q11	C	0.354	0.265	1.534	0.853	2.760	0.169	C	0.294	0.263	1.066	0.541	2.101	0.862	C	0.324	0.343	0.913	0.824	1.011	0.059	C	0.350	0.328	1.099	0.874	1.383	0.428

10	rs2802477	44696034	10q11	C	0.354	0.427	0.720	0.417	1.242	0.273	C	0.391	0.401	1.088	0.610	1.940	0.796	G	0.428	0.417	1.052	0.950	1.165	0.321	G	0.416	0.424	0.963	0.762	1.218	0.739
10	rs11594522	44696352	10q11	A	0.139	0.102	1.397	0.622	3.134	0.423	A	0.141	0.105	1.991	0.710	5.584	0.184	A	0.170	0.165	1.039	0.914	1.179	0.546	A	0.205	0.181	1.153	0.877	1.516	0.338
10	rs2054620	44698075	10q11	C	0.354	0.265	1.534	0.853	2.760	0.169	C	0.304	0.283	1.007	0.527	1.923	0.988	C	0.324	0.343	0.914	0.825	1.012	0.063	C	0.350	0.328	1.099	0.874	1.383	0.428
10	rs11238921	44699910	10q11	T	0.354	0.265	1.534	0.853	2.760	0.169	T	0.304	0.263	1.110	0.573	2.152	0.772	T	0.323	0.343	0.911	0.822	1.010	0.060	T	0.350	0.328	1.099	0.874	1.383	0.428
10	rs768676	44702681	10q11	A	0.076	0.092	0.849	0.355	2.031	0.814	A	0.120	0.105	1.065	0.428	2.651	0.848	A	0.067	0.058	1.166	0.956	1.423	0.125	A	0.056	0.063	0.880	0.547	1.418	0.585
10	rs3865770	44705969	10q11	T	0.139	0.102	1.397	0.622	3.134	0.423	T	0.141	0.105	1.991	0.710	5.584	0.184	A	0.170	0.165	1.039	0.914	1.179	0.546	A	0.205	0.181	1.153	0.877	1.516	0.338
10	rs1482473	44707598	10q11	C	0.160	0.143	1.090	0.515	2.307	0.819	C	0.109	0.158	0.496	0.213	1.158	0.109	C	0.144	0.166	0.847	0.743	0.967	0.016	C	0.137	0.139	0.987	0.711	1.369	0.952
10	rs3851257	44709171	10q11	T	0.229	0.167	1.330	0.739	2.393	0.356	T	0.163	0.204	0.656	0.343	1.257	0.220	T	0.323	0.343	0.912	0.823	1.011	0.059	T	0.347	0.330	1.077	0.857	1.354	0.530
10	rs12573558	44712128	10q11	A	0.139	0.102	1.397	0.622	3.134	0.423	A	0.141	0.105	1.991	0.710	5.584	0.184	A	0.179	0.178	1.005	0.887	1.138	0.924	A	0.210	0.191	1.119	0.854	1.467	0.442
10	rs11238935	44714402	10q11	T	0.099	0.122	0.736	0.327	1.658	0.461	T	0.065	0.099	0.373	0.125	1.117	0.071	T	0.099	0.119	0.815	0.699	0.950	0.008	T	0.087	0.077	1.150	0.763	1.732	0.487
10	rs2209067	44716469	10q11	A	0.207	0.143	1.571	0.776	3.179	0.198	A	0.196	0.132	1.801	0.785	4.132	0.150	A	0.179	0.179	1.001	0.884	1.134	0.981	A	0.211	0.191	1.130	0.862	1.481	0.406
10	rs1704219	44729958	10q11	C	0.347	0.255	1.527	0.848	2.752	0.182	C	0.294	0.276	0.978	0.513	1.865	0.909	C	0.308	0.328	0.912	0.821	1.012	0.068	C	0.336	0.314	1.100	0.872	1.388	0.443
10	rs7907961	44730995	10q11	C	0.243	0.133	2.166	1.054	4.451	0.044	C	0.228	0.178	1.589	0.750	3.365	0.259	C	0.209	0.209	0.998	0.887	1.124	0.966	C	0.248	0.237	1.062	0.824	1.369	0.632
10	rs1746043	44732825	10q11	C	0.347	0.255	1.527	0.848	2.752	0.182	C	0.294	0.276	0.978	0.513	1.865	0.909	C	0.310	0.333	0.895	0.807	0.994	0.026	C	0.336	0.312	1.108	0.878	1.398	0.421
10	rs647419	44734995	10q11	T	0.347	0.276	1.412	0.779	2.558	0.264	T	0.272	0.250	1.365	0.679	2.741	0.395	A	0.353	0.372	0.919	0.831	1.016	0.075	A	0.373	0.350	1.101	0.875	1.384	0.430
10	rs88796	44737036	10q11	C	0.319	0.255	1.312	0.737	2.335	0.370	C	0.250	0.237	0.938	0.469	1.875	0.854	C	0.267	0.290	0.889	0.797	0.990	0.021	C	0.284	0.249	1.193	0.930	1.530	0.200
10	rs617019	44737246	10q11	T	0.090	0.112	0.750	0.327	1.717	0.504	T	0.065	0.105	0.367	0.123	1.092	0.059	A	0.103	0.127	0.792	0.681	0.921	0.003	A	0.084	0.076	1.127	0.745	1.705	0.560
10	rs17155733	44737433	10q11	G	0.194	0.122	1.589	0.781	3.232	0.214	G	0.185	0.132	1.829	0.747	4.478	0.177	G	0.163	0.162	1.005	0.883	1.143	0.951	G	0.197	0.170	1.188	0.894	1.578	0.263
10	rs583489	44738688	10q11	C	0.208	0.184	1.129	0.597	2.136	0.725	C	0.120	0.132	0.643	0.288	1.433	0.254	G	0.111	0.138	0.779	0.673	0.902	0.003	G	0.090	0.084	1.099	0.736	1.641	0.648
10	rs676966	44739594	10q11	A	0.111	0.133	0.763	0.347	1.677	0.517	A	0.065	0.118	0.356	0.127	0.997	0.046	T	0.105	0.127	0.803	0.691	0.934	0.005	T	0.087	0.076	1.175	0.779	1.773	0.437
10	rs494207	44741256	10q11	T	0.208	0.184	1.129	0.597	2.136	0.725	T	0.120	0.132	0.643	0.288	1.433	0.254	A	0.111	0.138	0.779	0.672	0.902	0.003	A	0.092	0.084	1.121	0.752	1.672	0.570
10	rs541483	44746395	10q11	C	0.208	0.235	0.834	0.449	1.552	0.538	C	0.163	0.158	0.780	0.364	1.674	0.508	G	0.159	0.189	0.809	0.712	0.918	0.002	G	0.139	0.134	1.049	0.751	1.465	0.766
10	rs535176	44747059	10q11	A	0.264	0.276	0.930	0.527	1.641	0.792	A	0.163	0.171	0.718	0.345	1.496	0.365	T	0.159	0.189	0.809	0.712	0.918	0.002	T	0.139	0.134	1.049	0.751	1.465	0.766
10	rs622472	44749211	10q11	G	0.261	0.276	0.917	0.520	1.618	0.775	G	0.163	0.171	0.718	0.345	1.496	0.365	C	0.159	0.189	0.809	0.712	0.918	0.002	C	0.139	0.134	1.049	0.751	1.465	0.766
10	rs513391	44749708	10q11	G	0.264	0.276	0.930	0.527	1.641	0.792	G	0.163	0.171	0.718	0.345	1.496	0.365	C	0.159	0.190	0.807	0.710	0.917	0.002	C	0.139	0.134	1.049	0.751	1.465	0.766
10	rs11238956	44749854	10q11	C	0.299	0.327	0.896	0.520	1.542	0.703	C	0.304	0.322	0.984	0.516	1.875	0.920	C	0.348	0.329	1.085	0.981	1.200	0.095	C	0.311	0.345	0.860	0.681	1.085	0.226
10	rs687175	44751910	10q11	G	0.264	0.276	0.930	0.527	1.641	0.792	G	0.163	0.171	0.718	0.345	1.496	0.365	C	0.157	0.188	0.802	0.706	0.911	0.002	C	0.134	0.133	1.018	0.727	1.426	0.918
10	rs559580	44752078	10q11	C	0.204	0.235	0.818	0.439	1.522	0.523	C	0.156	0.158	0.750	0.348	1.618	0.464	C	0.155	0.187	0.794	0.698	0.903	0.002	C	0.129	0.128	1.017	0.722	1.432	0.931
10	rs559469	44752118	10q11	G	0.264	0.276	0.930	0.527	1.641	0.792	G	0.163	0.171	0.718	0.345	1.496	0.365	C	0.159	0.189	0.808	0.711	0.918	0.002	C	0.134	0.133	1.018	0.727	1.426	0.918
10	rs2437935	44752268	10q11	C	0.465	0.449	1.047	0.630	1.738	0.891	C	0.457	0.421	1.093	0.590	2.024	0.773	G	0.378	0.395	0.933	0.845	1.030	0.162	G	0.387	0.368	1.091	0.866	1.375	0.478
10	rs535949	44752330	10q11	A	0.271	0.271	0.992	0.566	1.738	0.956	A	0.163	0.187	0.706	0.344	1.447	0.339	T	0.157	0.188	0.803	0.707	0.913	0.002	T	0.134	0.133	1.018	0.727	1.426	0.918
10	rs671765	44752976	10q11	C	0.264	0.265	0.981	0.556	1.732	0.911	C	0.163	0.171	0.718	0.345	1.496	0.365	G	0.157	0.188	0.803	0.707	0.913	0.002	G	0.134	0.133	1.018	0.727	1.426	0.918
10	rs501120	44753867	10q11	G	0.268	0.276	0.946	0.536	1.671	0.847	G	0.163	0.171	0.718	0.345	1.496	0.365	C	0.157	0.188	0.803	0.707	0.913	0.002	C	0.134	0.133	1.018	0.727	1.426	0.918
10	rs579058	44755104	10q11	C	0.264	0.276	0.930	0.527	1.641	0.792	C	0.163	0.178	0.678	0.334	1.378	0.283	G	0.157	0.188	0.803	0.707	0.913	0.002	G	0.134	0.133	1.018	0.727	1.426	0.918
10	rs604674	44756894	10q11	A	0.264	0.276	0.930	0.527	1.641	0.792	A	0.163	0.178	0.678	0.334	1.378	0.283	T	0.157	0.188	0.803	0.707	0.913	0.002	T	0.134	0.133	1.018	0.727	1.426	0.918
10	rs487465	44758197	10q11	G	0.264	0.276	0.930	0.527	1.641	0.792	G	0.163	0.178	0.678	0.334	1.378	0.283	C	0.157	0.188	0.803	0.707	0.913	0.002	C	0.134	0.133	1.018	0.727	1.426	0.918
10	rs475926	44760887	10q11	C	0.380	0.354	1.078	0.623	1.866	0.783	C	0.294	0.273	0.951	0.497	1.818	0.875	G	0.309	0.334	0.890	0.803	0.987	0.025	G	0.329	0.303	1.128	0.892	1.427	0.338
10	rs1632484	44773984	10q11	A	0.229	0.255	0.843	0.467	1.523	0.593	A	0.163	0.171	0.687	0.337	1.399	0.302	T	0.152	0.183	0.799	0.703	0.910	0.002	T	0.131	0.126	1.048	0.744	1.476	0.801
10	rs1746048	44775824	10q11	T	0.264	0.276	0.930	0.527	1.641	0.792	T	0.163	0.171	0.687	0.337	1.399	0.302	T	0.154	0.186	0.797	0.701	0.906	0.002	T	0.132	0.133	1.003	0.716	1.406	0.985
10	rs1746049	44776310	10q11	T	0.264	0.276	0.930	0.527	1.641	0.792	T	0.163	0.171	0.687	0.337	1.399	0.302	T	0.152	0.183	0.800	0.703	0.910	0.002	T	0.132	0.131	1.018	0.727	1.427	0.923
10	rs1746052	44778546	10q11	C	0.174	0.225	0.726	0.383	1.379	0.328	C	0.087	0.145	0.416	0.175	0.989	0.034	C	0.148	0.177	0.807	0.708	0.919	0.002	C	0.131	0.128	1.034	0.736	1.452	0.861
10	rs800314	44786364	10q11	G	0.063	0.112	0.470	0.176	1.250	0.116	G	0.043	0.086	0.262	0.075	0.917	0.022	G	0.076	0.096	0.778	0.656	0.923	0.005	G	0.073	0.066	1.114	0.712	1.743	0.652
10	rs11598523	44791433	10q11	A	0.120	0.102	1.130	0.499	2.561	0.779	A	0.120	0.099	1.754	0.601	5.118	0.282	A	0.164	0.156	1.055	0.926	1.201	0.415	A	0.208	0.174	1.247	0.942	1.650	0.135
10	rs2505734	44793299	10q11	C	0.438	0.417	1.062	0.632	1.787	0.833	C	0.457	0.434	0.970	0.536	1.755	0.920	C	0.360	0.379	0.922	0.835	1.019	0.111	C	0.358	0.334	1.110	0.882	1.398	0.381
10	rs754713	44797087	10q11	T	0.375	0.313	1.288	0.757	2.191	0.368	T	0.413	0.322	1.618	0.880	2.975	0.114	T	0.283	0.283	1.002	0.901	1.114	0.970	T	0.284	0.267	1.089	0.854	1.388	0.492
10	rs800320	44798482	10q11	T	0.076	0.092	0.855	0.340	2.147	0.757	T	0.163	0.151	1.182	0.530	2.640	0.720	T	0.047	0.040	1.176	0.931	1.486	0.184	T	0.032	0.033	0.969	0.514	1.826	0.916
10	rs800323	44801673	10q11	G	0.340	0.316	1.086	0.643	1.836	0.788	G	0.413	0.355	1.326	0.729	2.410	0.342	G	0.283	0.284	0.995	0.894	1.106	0.924	G	0.287	0.267	1.106	0.867	1.411	0.405
10	rs2437934	44803925	10q11	G	0.438	0.429	1.011	0.605	1.688	0.969	G	0.457	0.434	0.970	0.536	1.755	0.920	G	0.358	0.378	0.920	0.833	1.016	0.106	G	0.360	0.334	1.117	0.888	1.406	0.347
10	rs11238983	44810205	10q11	A	0.217	0.192	1.138	0.576	2.250	0.711	A	0.239	0.165	1.720	0.807	3.665	0.164	A	0.156	0.174	0.876	0.769	0.998	0.041	A	0.119	0.151	0.764	0.553	1.056	0.099
10	rs2146807	44813738	10q11	C	0.167	0.163	1.003	0.485	2.076	0.992	C	0.239	0.132	2.644	1.121	6.237	0.039	C	0.155	0.173	0.879	0.771	1.002	0.049	C	0.119	0.158	0.717	0.516	0.996	0.043
10	rs2146808	44813777	10q11	C	0.059	0.092	0.620	0.219	1.757	0.387	C	0.152	0.138	1.267	0.560	2.864	0.601	C	0.043	0.039	1.102	0.867	1.400	0.422	C	0.031	0.030	1.019	0.529	1.965	0.958
10	rs7082209	44814336	10q11	G	0.201	0.184	1.097	0.561	2.146	0.809	G	0.239	0.165	1.720	0.807	3.665	0.164	G	0.159	0.176	0.881	0.773	1.003	0.044	G	0.116	0.153	0.729	0.526	1.012	0.047
10	rs800310	44815048	10q11	A	0.306	0.296	1.031	0.593	1.795	0.970	A	0.348	0.290	1.559	0.802	3.029	0.198	A	0.224	0.240	0.914	0.815	1.026	0.123	A	0.155	0.204	0.707	0.524	0.955	0.017
10	rs2505735	44815709	10q11	A	0.285	0.286	0.983	0.542	1.784	0.895	A	0.300	0.287	1.004	0.524	1.925	0.997	A	0.203	0.217	0.917	0.814	1.033	0.139	A	0.150	0.181	0.793	0.585	1.075	0.132
10	rs977754	44817419	10q11	G	0.257	0.225	1.194	0.647	2.203	0.640	G	0.283	0.250	1.048	0.533	2.059	0.902	G	0.191	0.203	0.922	0.816	1.041	0.183	G	0.134	0.172	0.736	0.535	1.012	0.064
10	rs812889	44818563	10q11	G	0.076	0.071	1.118	0.420	2.976	0.825	G	0.087	0.086	0.760	0.272	2.129	0.585	G	0.089	0.087	1.030	0.869	1.221	0.748	G	0.053	0.074	0.709	0.448	1.122	0.130
10	rs2476351	44820157	10q11	C	0.229	0.194	1.223	0.648	2.309	0.565	C	0.272	0.204	1.344	0.667	2.709	0.423	C	0.185	0.198	0.921	0.814	1.041	0.171	C	0.129	0.161	0.768	0.557	1.060	0.123
10	rs11238987	44821220	10q11	A	0.069	0.102	0.598	0.236	1.516	0.286	A	0.065	0.087	0.576	0.211	1.569	0.287	A	0.029	0.036	0.798	0.611	1.042	0.096	A	0.034	0.030	1.139	0.600	2.163	0.724
10	rs10508884	44821387	10q11	T	0.285	0.286	0.950	0.515	1.752	0.861	T	0.294	0.355	0.737	0.397	1.368	0.312	T	0.272	0.268	1.021	0.917	1.137	0.683	T	0.303	0.276	1.141	0.895	1.454	0.289
10	rs1111259	44821772	10q11	T	0.132	0.102	1.236	0.561	2.724	0.582	T	0.174	0.125	1.702	0.717	4.041	0.190	T	0.106	0.121	0.860	0.740	1.000	0.042	T	0.079	0.091	0.844	0.563	1.265	0.436
10	rs2505741	44823136	10q11	A	0.424	0.439	0.995	0.571	1.732	0.995	A	0.402	0.388	1.051	0.596	1.853	0.890	G	0.472	0.480	0.969	0.880	1.066	0.527	G	0.453	0.443	1.043	0.837	1.299	0.704
10	rs2028100	44826376	10q11	A	0.257	0.265	0.906	0.479	1.714	0.739	A	0.228	0.303	0.609	0.312	1.190	0.177	A	0.249	0.245	1.019	0.913	1.138	0.740	A	0.290	0.254	1.198	0.936	1.532	0.162
10	rs1836982	44827164	10q11	G	0.264	0.235	1.138	0.633	2.048	0.657	G	0.348	0.250	1.797	0.916	3.524	0.099	G	0.209	0.218	0.947	0.841	1.065	0.361	G	0.144	0.175	0.777	0.567	1.066	0.125
10	rs982097	44828234	10q11	T	0.493	0.500	1.022	0.596	1.752	0.939	T	0.424	0.447	0.942	0.534	1.662	0.809	G	0.461	0.466	0.977	0.888	1.075	0.635	G	0.436	0.432	1.015	0.815	1.264	0.882
10	rs7918046	44830727	10q11	T	0.299	0.276	1.075	0.604	1.913	0.774	T	0.326	0.336	0.945	0.524	1.704	0.805	T	0.293	0.299	0.975	0.879	1.081	0.615	T	0.284	0.279	1.023	0.806	1.298	0.852
10	rs11815919	44831379	10q11	T	0.167	0.174	0.939	0.468	1.882	0.862	T	0.152	0.211	0.574	0.256	1.288	0.193	T	0.188	0.179	1.062	0.938	1.201	0.324	T	0.203	0.186	1.111	0.847	1.458	0.448
10	rs928565	44832884	10q11	T	0.430	0.439	1.008	0.597	1.701	0.970	T	0.402	0.375	1.215	0.688	2.147	0.475	A	0.493	0.487	1.025	0.931	1.128	0.632	G	0.466	0.476	0.963	0.773	1.200	0.727
10	rs11599561	44833031	10q11	C	0.090	0.102	0.884	0.367	2.126	0.804	C	0.043	0.105	0.204	0.056	0.740	0.011	C	0.106	0.112	0.939	0.804	1.096	0.419	C	0.113	0.110	1.025	0.718	1.465	0.918
10	rs266080	44833894	10q11	G	0.063	0.061	1.006	0.357	2.837	0.979	G	0.087	0.033	6.354	1.398	#####	0.003	G	0.024	0.020	1.190	0.862	1.642	0.300	G	0.013	0.021	0.624	0.255	1.527	0.325
10	rs1144482	44835963	10q11	A	0.389	0.388	1.066	0.620	1.833	0.829	A	0.380	0.342	1.197	0.679	2.109	0.530	T	0.480	0.473	1.031	0.936	1.135	0.529	C	0.482	0.487	0.983	0.789	1.224	0.876
10	rs1360724	44837267	10q11	G	0.299	0.286	1.018	0.572	1.812	0.949	G	0.326	0.336	0.945	0.524	1.704	0.805	G	0.317	0.320	0.985	0.889	1.091	0.765	G	0.307	0.300	1.033	0.817	1.306	0.796
10	rs10900025	44838019	10q11	G	0.431	0.429	0.951	0.554	1.633	0.863	G	0.424	0.474	0.770	0.438	1.355	0.401	G	0.402	0.413	0.955	0.867	1.052	0.362	G	0.407	0.390	1.070	0.858	1.334	0.581
10	rs7069891	44838464	10q11	T	0.099	0.102	0.966	0.406	2.299	0.986	T	0.043	0.112	0.201	0.056	0.725	0.010	T	0.107	0.112	0.945	0.810	1.103	0.478	T	0.113	0.110	1.025	0.718	1.465	0.918
10	rs77839	44838530	10q11	G	0.486	0.490	0.927	0.540	1.592	0.792	G	0.511	0.507	1.022	0.582	1.795	0.889	G	0.426	0.433	0.969	0.880	1.067	0.535	G	0.419	0.410	1.038	0.834	1.293	0.742
10	rs17390084	44839865	10q11	A	0.042	0.073	0.536	0.167	1.715	0.268	A	0.076	0.072	0.794	0.244	2.582	0.738	A	0.041	0.043	0.934	0.734	1.189	0.576	A	0.040	0.028	1.436	0.777	2.653	0.248
10	rs10793536	44842048	10q11	C	0.472	0.480	0.905	0.528	1.552	0.712	C	0.446	0.507	0.789	0.455	1.369	0.402	C	0.433	0.445	0.951	0.864	1.048	0.327	C	0.426	0.413	1.051	0.844	1.308	0.683
10	rs266076	44844381	10q11	A	0.472	0.459	1.141	0.656	1.983	0.629	G	0.522	0.540	1.004	0.579	1.742	0.984	G	0.435	0.446	0.959	0.871	1.055	0.397	G	0.426	0.415	1.044	0.839	1.299	0.718
10	rs1144480	44848921	10q11	A	0.410	0.418	1.027	0.604	1.748	0.927	A	0.370	0.329	1.191	0.668	2.124	0.557	A	0.463	0.456	1.029	0.935	1.134	0.579	T	0.497	0.503	0.978	0.787	1.215	0.841
10	rs266109	44850424	10q11	G	0.169	0.133	1.268	0.619	2.596	0.532	G	0.196	0.138	1.758	0.769	4.018	0.165	G	0.116	0.128	0.893	0.771	1.033	0.117	G	0.097	0.104	0.920	0.640	1.324	0.665
10	rs1144477	44851737	10q11	A	0.424	0.439	0.995	0.589	1.681	0.996	A	0.424	0.349	1.423	0.793	2.554	0.245	T	0.482	0.473	1.037	0.942	1.142	0.482	C	0.489	0.489	1.001	0.806	1.245	0.993
10	rs266105	44855663	10q11	A	0.194	0.102	2.134	0.976	4.666	0.064	A	0.185	0.118	2.241	0.975	5.152	0.057	A	0.137	0.140	0.974	0.849	1.118	0.681	A	0.113	0.110	1.026	0.726	1.450	0.881
10	rs11595588	44855740	10q11	C	0.528	0.417	1.554	0.919	2.630	0.096	C	0.421	0.426	1.103	0.618	1.969	0.739	C	0.395	0.399	0.984	0.892	1.085	0.716	C	0.390	0.386	1.015	0.817	1.262	0.862
10	rs17391002	44855927	10q11	G	0.278	0.255	1.110	0.611	2.017	0.739	G	0.196	0.237	0.743	0.372	1.484	0.391	G	0.203	0.196	1.048	0.928	1.183	0.464	G	0.239	0.229	1.050	0.819	1.346	0.710
10	rs266103	44856370	10q11	T	0.215	0.133	1.854	0.896	3.836	0.101	T	0.196	0.171	1.944	0.885	4.272	0.093	T	0.143	0.148	0.958	0.838	1.096	0.529	T	0.111	0.110	1.010	0.716	1.424	0.957
10	rs185545	44858840	10q11	C	0.278	0.225	1.418	0.741	2.711	0.269	C	0.261	0.204	1.609	0.812	3.191	0.166	C	0.185	0.181	1.032	0.910	1.170	0.620	C	0.168	0.194	0.849	0.643	1.121	0.225
10	rs7918568	44861220	10q11	T	0.306	0.235	1.409	0.790	2.515	0.244	T	0.294	0.191	1.954	0.932	4.097	0.086	T	0.175	0.166	1.066	0.940	1.209	0.342	T	0.186	0.186	0.996	0.751	1.322	0.974
10	rs7915848	44863434	10q11	C	0.285	0.235	1.294	0.715	2.342	0.449	C	0.272	0.184	1.906	0.875	4.151	0.120	C	0.176	0.168	1.061	0.936	1.203	0.366	C	0.184	0.183	1.005	0.755	1.338	0.973
10	rs266094	44864300	10q11	T	0.141	0.133	1.112	0.541	2.286	0.779	T	0.163	0.105	1.852	0.753	4.557	0.153	T	0.143	0.150	0.951	0.831	1.089	0.439	T	0.124	0.126	0.984	0.709	1.364	0.902
10	rs266093	44866208	10q11	C	0.479	0.429	1.241	0.759	2.027	0.381	C	0.533	0.388	2.084	1.086	4.001	0.026	C	0.362	0.359	1.014	0.918	1.120	0.786	C	0.339	0.344	0.979	0.775	1.237	0.874
10	rs1029153	44867146	10q11	C	0.208	0.265	0.738	0.409	1.330	0.329	C	0.228	0.263	0.836	0.397	1.762	0.642	G	0.274	0.279	0.976	0.878	1.086	0.660	G	0.284	0.273	1.059	0.825	1.361	0.637
10	rs1801157	44868257	10q11	A	0.167	0.194	0.821	0.429	1.574	0.482	A	0.152	0.243	0.368	0.153	0.889	0.026	T	0.245	0.247	0.989	0.885	1.106	0.855	T	0.216	0.216	0.999	0.760	1.312	0.985
10	rs266089	44869427	10q11	A	0.139	0.133	1.082	0.528	2.217	0.823	A	0.185	0.145	1.343	0.608	2.966	0.465	A	0.150	0.157	0.950	0.831	1.085	0.412	A	0.132	0.148	0.879	0.641	1.206	0.426
10	rs266088	44870015	10q11	T	0.153	0.112	1.378	0.659	2.882	0.401	T	0.130	0.151	0.808	0.348	1.877	0.573	T	0.150	0.144	1.050	0.918	1.201	0.448	T	0.187	0.185	1.017	0.764	1.353	0.923
10	rs266087	44871062	10q11	A	0.340	0.347	0.952	0.551	1.645	0.835	A	0.304	0.428	0.400	0.194	0.827	0.014	A	0.402	0.403	0.994	0.901	1.095	0.900	A	0.413	0.410	1.010	0.802	1.274	0.930
10	rs2297630	44871548	10q11	A	0.153	0.204	0.681	0.346	1.339	0.268	A	0.141	0.165	0.902	0.373	2.179	0.808	A	0.273	0.280	0.965	0.867	1.073	0.503	A	0.282	0.273	1.050	0.818	1.350	0.693
10	rs2839690	44875166	10q11	C	0.285	0.235	1.294	0.715	2.342	0.449	C	0.283	0.197	1.611	0.764	3.396	0.211	G	0.177	0.167	1.070	0.943	1.215	0.304	G	0.181	0.186	0.964	0.723	1.285	0.786
10	rs3780891	44878713	10q11	A	0.097	0.061	1.644	0.604	4.474	0.341	A	0.065	0.099	0.653	0.224	1.899	0.516	A	0.084	0.074	1.152	0.961	1.380	0.144	A	0.105	0.109	0.959	0.672	1.369	0.821

F_A, allele frequency in cases (Affected); F_U, allele frequency in controls (Unaffected); OR, odd ratio for the reference allele (A1); L_95, lower bound of 95% confidence interval for odds ratio; U_95, upper bound of 95% confidence interval for odds ratio; P, p-value after a permutation test (1,000 permutations).

Genomic location of the genetic variants.
