# Supplementary material for: Analysis of Genomic Regions Associated With Coronary Artery Disease Reveals Continent-Specific Single Nucleotide Polymorphisms in North African Populations
Source: J Epidemiol. 2016 May 5;26(5):264–71. doi: 10.2188/jea.JE20150034 (PMC4848325; doi:10.2188/jea.JE20150034)
Supplement: eTable 3. [file je-26-264-s003.pdf]

**eTable 3.** Genomic location of the 6 North African SNPs used to calculate the risk score in the case-control samples

| Region | Position | SNP |
|---|---|---|
| 9p21 | 22136489 | rs1333051 |
| 9p21 | 22191189 | rs828576 |
| 10q11 | 44730995 | rs7907961 |
| 10q11 | 44786364 | rs800314 |
| 10q11 | 44856370 | rs266103 |
| 10q11 | 44861220 | rs7918568 |

SNP, single nucleotide polymorphism.
