# Supplementary material for: Analysis of Genomic Regions Associated With Coronary Artery Disease Reveals Continent-Specific Single Nucleotide Polymorphisms in North African Populations
Source: J Epidemiol. 2016 May 5;26(5):264–71. doi: 10.2188/jea.JE20150034 (PMC4848325; doi:10.2188/jea.JE20150034)
Supplement: eFigure 2B. [file je-26-264-s006.pdf]

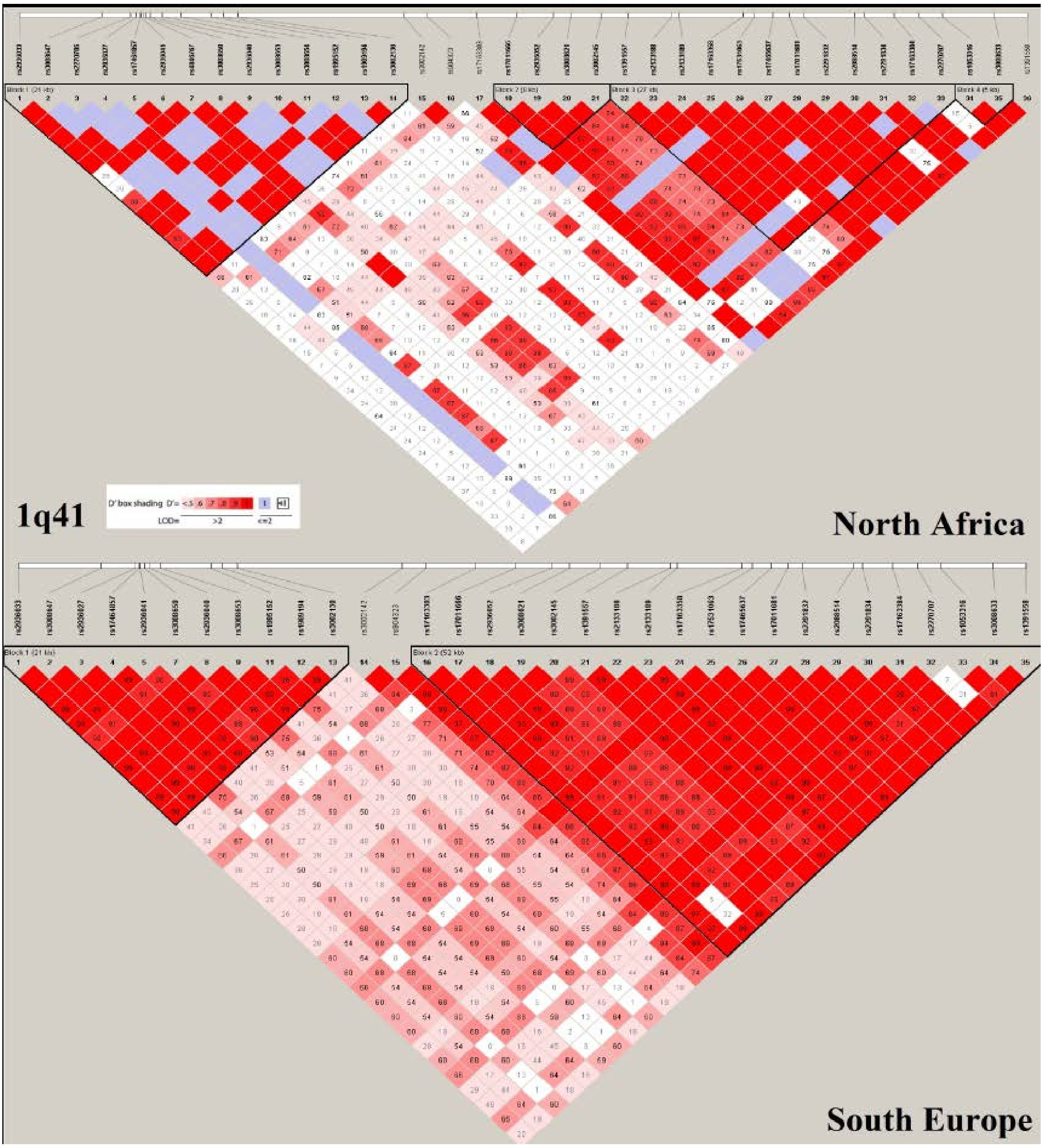

eFigure 2B. Linkage disequilibrium patterns and haplotype block structure observed in North Africa and in southern Europe for the region 1q41
