# Supplementary material for: Analysis of Genomic Regions Associated With Coronary Artery Disease Reveals Continent-Specific Single Nucleotide Polymorphisms in North African Populations
Source: J Epidemiol. 2016 May 5;26(5):264–71. doi: 10.2188/jea.JE20150034 (PMC4848325; doi:10.2188/jea.JE20150034)
Supplement: eFigure 2C. [file je-26-264-s007.pdf]

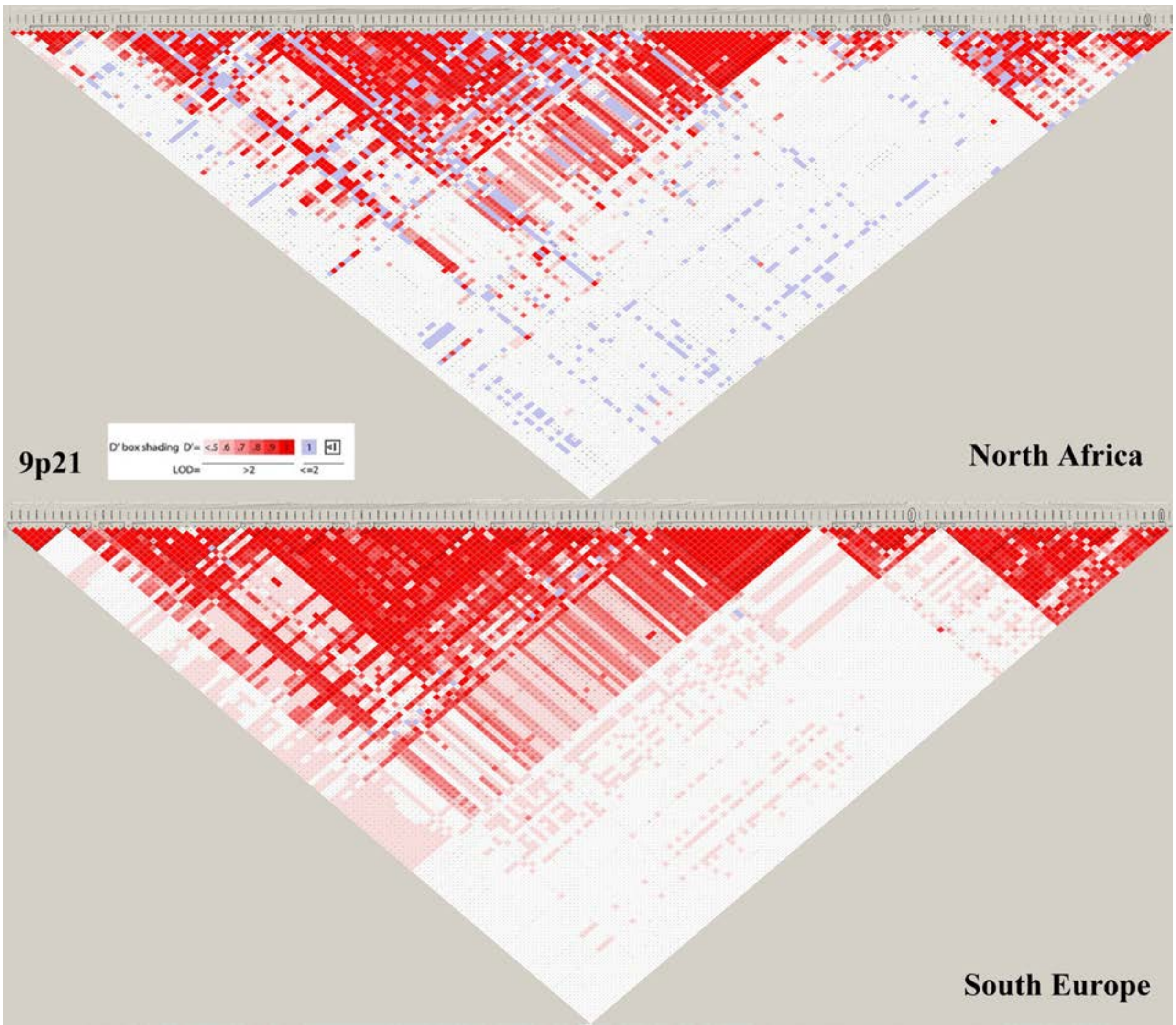

eFigure 2C. LD patterns and haplotype block structure observed in North Africa and in South Europe for the region 9p21. LD colors and values are based on D'. The SNPs associated in the North Africa meta-analysis are marked with a black circle.
