# Supplementary material for: Analysis of Genomic Regions Associated With Coronary Artery Disease Reveals Continent-Specific Single Nucleotide Polymorphisms in North African Populations
Source: J Epidemiol. 2016 May 5;26(5):264–71. doi: 10.2188/jea.JE20150034 (PMC4848325; doi:10.2188/jea.JE20150034)
Supplement: eFigure 3. [file je-26-264-s009.pdf]

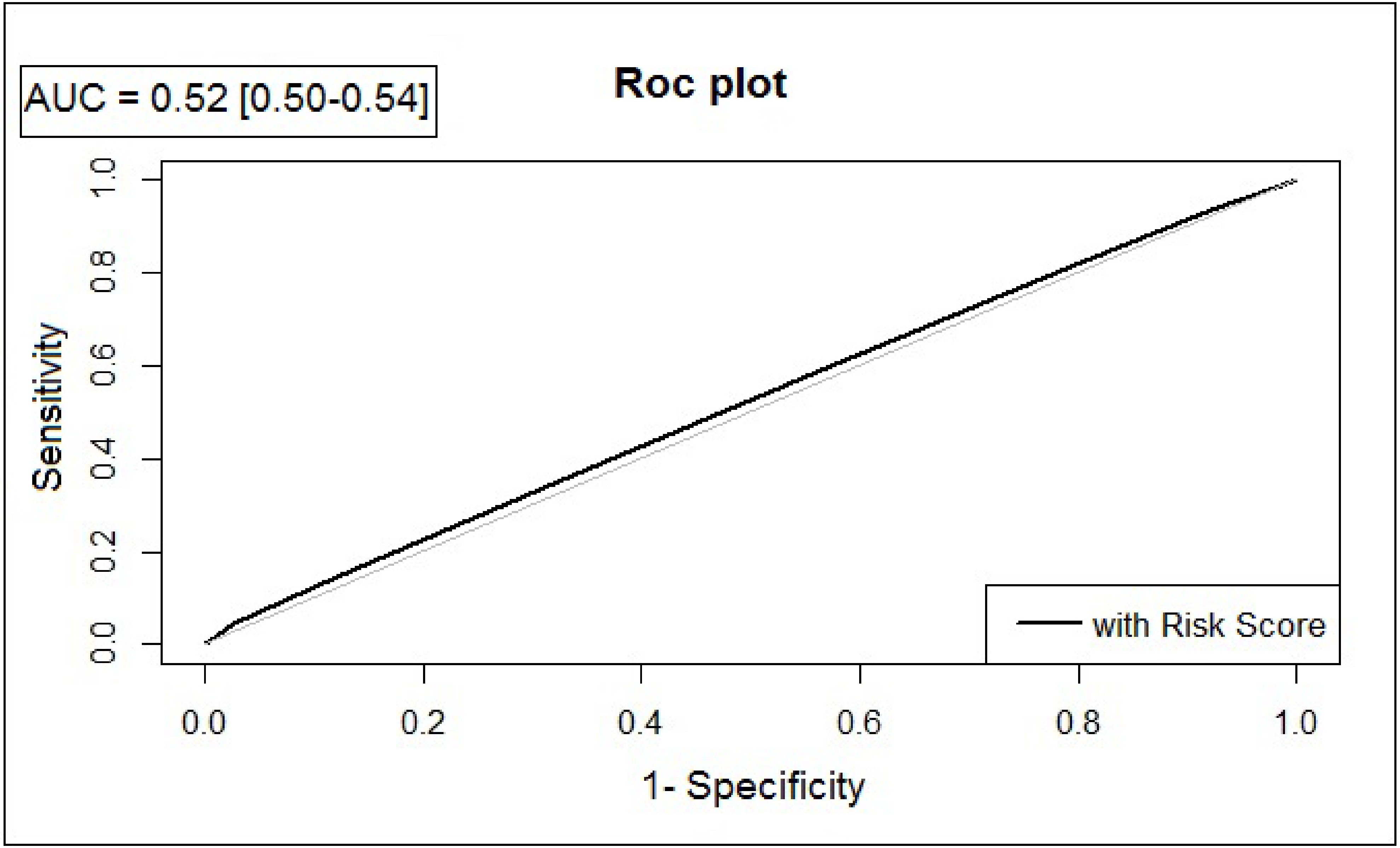

eFigure 3. Receiver operating characteristic (ROC) curves in South Europe. The number of risk alleles was based on the risk alleles associated with CAD in North Africans. AUC: Area Under the ROC Curve with 95% confidence interval.
